# Somatic 9p24.1 alterations in HPV^–^ head and neck squamous cancer dictate immune microenvironment and anti-PD-1 checkpoint inhibitor activity

**DOI:** 10.1073/pnas.2213835119

**Published:** 2022-11-17

**Authors:** Xin Zhao, Ezra E. W. Cohen, William N. William, Joy J. Bianchi, Jim P. Abraham, Daniel Magee, David B. Spetzler, J. Silvio Gutkind, Ludmil B. Alexandrov, Webster K. Cavenee, Scott M. Lippman, Teresa Davoli

**Affiliations:** ^a^Department of Biochemistry and Molecular Pharmacology, Institute for Systems Genetics, New York University Langone Health, New York, NY 10016;; ^b^Moores Cancer Center, University of California San Diego, La Jolla, CA 92037;; ^c^Department of Medicine, University of California San Diego, La Jolla, CA 92037;; ^d^Thoracic/Head and Neck Medical Oncology, The University of Texas MD Anderson Cancer Center, Houston, TX 77030;; ^e^Hospital BP, a Beneficência Portuguesa de São Paulo, 01323-001 São Paulo, Brazil;; ^f^Research and Development, Caris Life Sciences, Irving, TX 75039;; ^g^Department of Pharmacology, University of California San Diego, La Jolla, CA 92037;; ^h^Department of Cellular and Molecular Medicine, University of California San Diego, La Jolla, CA 92037;; ^i^Department of Bioengineering, University of California San Diego, La Jolla, CA 92037;; ^j^Ludwig Institute for Cancer Research, University of California San Diego, La Jolla, CA 92037

**Keywords:** 9p21, 9p24, immunotherapy, genomics, head neck cancer

## Abstract

Somatic copy number alterations (SCNAs), generally (1) losses containing interferons and interferon-pathway genes, many on chromosome 9p, predict immune-cold, immune checkpoint therapy (ICT)-resistant tumors (2); however, genomic regions mediating these effects are unclear and probably tissue specific. Previously, 9p21.3 loss was found to be an early genetic driver of human papillomavirus–negative (HPV^–^) head and neck squamous cancer (HNSC), associated with an immune-cold tumor microenvironment (TME) signal, and recent evidence suggested that this TME-cold phenotype was greatly enhanced with 9p21 deletion size, notably encompassing band 9p24.1 (3). Here, we report multi-omic, -threshold and continuous-variable dissection of 9p21 and 9p24 loci (including depth and degree of somatic alteration of each band at each locus, and each gene at each band) and TME of four HPV^–^ HNSC cohorts. Preferential 9p24 deletion, CD8 T-cell immune-cold associations were observed, driven by 9p24.1 loss, and in turn by an essential telomeric regulatory gene element, *JAK2-CD274*. Surprisingly, same genetic region gains were immune hot. Related 9p21-TME analyses were less evident. Inherent 9p-band-level influences on anti-PD1 ICT survival rates, coincident with TME patterns, were also observed. At a 9p24.1 whole-transcriptome expression threshold of 60th percentile, ICT survival rate exceeded that of lower expression percentiles and of chemotherapy; below this transcript threshold, ICT survival was inferior to chemotherapy, the latter unaffected by 9p24.1 expression level (*P-*values < 0.01, including in a PD-L1 immunohistochemistry-positive patient subgroup). Whole-exome analyses of 10 solid-tumor types suggest that these 9p-related ICT findings could be relevant to squamous cancers, in which 9p24.1 gain/immune-hot associations exist.

## Significance

Despite remarkable advances in immune-checkpoint therapy (ICT) for human papillomavirus–negative (HPV^–^) head and neck squamous cancer (HNSC), drug resistance remains prevalent, poorly understood, and largely unidentified by existing biomarker tests. Somatic alterations of interferon or interferon-pathway genes, many on chromosome 9p, predict immune-cold, ICT-resistant tumors; genomic regions mediating these effects, however, are unclear and likely tissue specific. Multiomic analyses of HPV^–^ HNSC cohorts identified preferential 9p24.1–immune oncology (IO) associations: copy-number losses with immune-cold, ICT-resistant and gains with immune-hot, ICT-responsive disease. At a 9p24.1 expression threshold of 60th percentile, ICT median survival was 3-fold higher than chemotherapy; below this threshold, chemotherapy survival rates exceeded ICT. These 9p-IO findings reveal novel genetically defined ICT resistance and sensitivity in HPV^–^ squamous tumors.

Anti-PD-1 immune checkpoint therapy (ICT) therapy is an integral part of the standard of care in head and neck squamous cell cancer (HNSC) (4). The definitive demonstrations of improved efficacy came through randomized trials, initially in the recurrent/metastatic setting after platinum failure with anti-PD-1 antibodies nivolumab and pembrolizumab (5, 6). These studies demonstrated improved overall survival with anti-PD-1 therapy compared to chemotherapy or cetuximab. Subsequently, KEYNOTE-048 tested either pembrolizumab monotherapy or pembrolizumab and chemotherapy against a triplet regimen of platinum/fluorouracil/cetuximab in first-line recurrent/metastatic disease (7). This study demonstrated improved survival of pembrolizumab monotherapy in patients whose tumors expressed PD-L1 protein by immunohistochemistry. Despite remarkable deep and durable responses, the majority of patients do not benefit from anti-PD-1 therapy, even those whose tumors express high levels of PD-L1 (8). Furthermore, in ∼20% of patients with no tumor PD-L1 expression treated with pembrolizumab alone, overall survival is worse compared to chemotherapy (9). It is clear that ICT-responsive tumors demonstrate evidence of an antitumor immune response probably related to local interferon-γ (IFN-γ) release; *CD274* (which encodes PD-L1) is an IFN-γ-responsive gene. Evidence of this IFN-γ antitumor immune response includes associations with CD8 T-cell infiltration, cytotoxic immune score, gene expression profiles, and PD-L1 protein expression (10). Although not a companion diagnostic for HNSC, the latter is most widely used in clinical practice because of its simplicity and the fact that other assays have not proven to be more predictive. Genomic-based findings have been evaluated as candidate biomarkers of ICT benefits, orthogonal to biomarkers dependent on an IFN-γ response. The most widely studied of these is tumor mutational burden, first reported to be elevated in HNSC and bladder and lung cancers (11). Pembrolizumab has been approved by the US Food and Drug Administration (FDA) for all cancers with a tumor mutational burden of ≥10 mutations/megabase, based on clinical trials with limited HNSC representation (12). Another tumor agnostic genomic biomarker that has garnered FDA approval for anti-PD-1 antibodies is mismatch repair defects (13, 14), rarely present in HNSC. Although immunogenomic studies have variably identified specific genomic/pathway alterations associated with resistance to ICT in diverse tumors and model systems (15–18), none are validated in HNSC for use in standard clinical practice. Therefore, there is an urgent unmet medical need to elucidate mechanisms of resistance and improved predictive biomarkers to identify patient subpopulations likely to respond to ICT, in order to optimize the likelihood of therapeutic success and reduce the immune oncology (IO)-related adverse event risks and expense of unnecessary treatment.

**Fig. 1.**
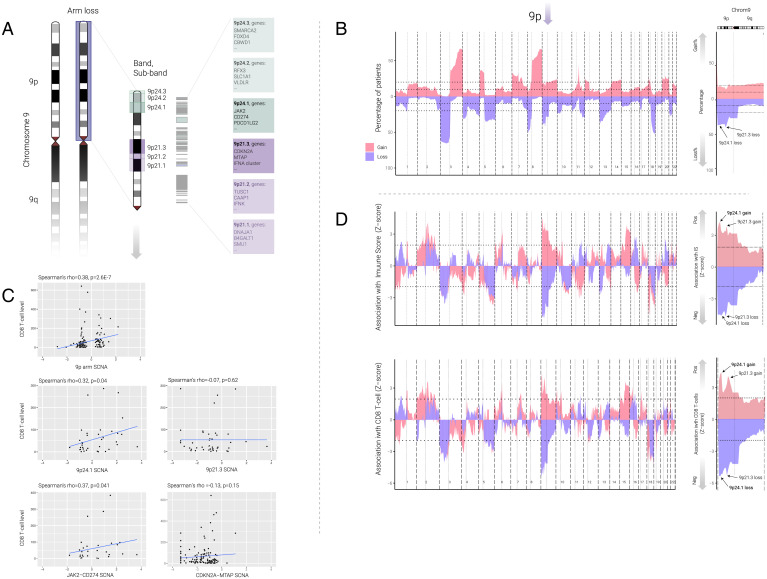
Association between SCNAs and immune infiltrates on HPV^–^ HNSC. (*A*) Schematics of chromosome 9p, 9p24, and 9p21 loci and the three bands at each locus. Different intensity of green color indicates different bands on 9p24 locus (9p24.1, 9p24.2, and 9p24.3) with related example genes, and different intensity of purple color indicates different bands on 9p21 locus (9p21.1, 9p21.3, and 9p21.3) with related example genes on each cytoband. (*B*) *Left*: Area plot represents the percentage of patients with gains (red) or losses (blue) for HPV^–^ HNSC for each chromosomal region. Each chromosome is split by a bold vertical dotted line, and the p and q arms are split by the light vertical dotted line. The horizontal dotted line represents 10% and 20% of the patients. *Right*: Detailed plot on chromosome 9 only; black text with arrow marks the location of 9p24.1 and 9p21.3. The p and q arms are split by the light vertical dotted line, and the horizontal dotted lines represent 10% and 20%. (*C*) Dot plot represents correlation between SCNA (focal level, continuous variable) and CD8 T-cell level. *Top Left*: Correlation between 9p arm-level SCNA and CD8 T-cell level; each black dot represents each patient. *Middle Left*: Correlation between 9p24.1 SCNA and CD8 T-cells level. *Top Right*: Correlation between 9p21.3 focal SCNA and CD8 T-cell level. *Bottom Left*: Correlation between *JAK2*-*CD274* SCNA and CD8 T-cell level. *Bottom Right*: Correlation between *CDKN2A*-*MTAP* SCNA and CD8 T-cell level. Spearman’s correlation coefficients and related *P* values are indicated in the top of the plot. (*D*) Area plot represents the association (*z*-score) between gains (red) and losses (blue) and immune score (*Top*) or CD8 T-cell level (*Bottom*) across each chromosome in multivariable logistic regression model (for example, immune score ∼9p loss + SCNA level). Each chromosome is split by a bold vertical dotted line, and the p and q arms are split by the light vertical dotted line. The horizontal dotted line represents *P* = 0.05. Positive *z*-score indicates the SCNA is positively associated with the immune score or CD8 T-cell level, negative *z*-score indicates the SCNA is negatively associated with the immune score or CD8 T-cell level. Arrow above 9p indicates the location of 9p. High-power images (*Right*) are focused on the chromosome 9; text with black arrow indicates the location of 9p21.3 and 9p24.1. Red indicates gains and blue indicates losses.

Somatic copy number alterations (SCNAs), central chromosomal events in most cancers, can increase or decrease the dosage of specific genomic regions. Deletions of 9p21.3 band (19) and 9p arm (20), are among the most frequent recurrent SCNA events in human cancer, and have been implicated in tumor initiation, evolution, and progression through cell cycle and tumor metabolism regulation. More recently, SCNAs, notably losses containing IFNs and IFN pathway genes, many on chromosome 9p, have been reported to predict immune-cold, immune checkpoint therapy (ICT)-resistant tumors (1, 21). Immunogenetic studies of 9p21.3 copy number alterations, focused primarily on *CDKN2A* deletion (22, 23), can encompass a cluster of 16 type-I IFN genes (Fig. 1*A*) involved in antitumor immune responses (24), while IFN-γ pathway gene alterations at 9p24.1 have been reported to correlate with immune-cold, ICT-resistant tumors, primarily in metastatic melanoma (25). Notably associated with 9p24.1 ICT resistance are loss-of-function mutations in *JAK2* and IFN-γ resistance in cell lines lacking *JAK2* (18). These findings were extended by reports of overall SCNA and copy-number loss (but not gain) burden, including 9p, associated with dual PD-1 and CTLA4 checkpoint-inhibitor resistant metastatic melanoma (1, 21).

Previously, we identified recurrent 9p21.3 loss as an early genetic driver of human papillomavirus-negative (HPV^–^) HNSC (26), associated with an immune-cold tumor microenvironment (TME) signal limited to HPV^–^ disease (2). Recent studies confirmed these 9p21.3 deletion/IO observations, also limited to HPV^–^ HNSC, including CD8 T-cell depletion and *CXCL9/10* suppression, extended these findings to several other solid tumors, and suggested that codeletions extending to 9p24.1 were important to the immune-cold TME phenotype (3). These studies gave rise to new questions as to which genetic regions/genes on 9p are the main drivers of the immune-cold phenotype, along with the underlying molecular mechanism. The 9p24.1 region harbors the IFN-γ-related gene *JAK2*, as well as both ligands of *PDCD1* (also known as PD-1)—*CD274* (encoding PD-L1) and *PDCD1LG2* (encoding PD-L2)—targets of PD-1/-L1 axis inhibitors (Fig. 1*A*), and whose codeletions have not been fully characterized in immune oncology. In contrast, amplifications of genes at 9p24.1 have been associated with an increased abundance of both PD-1 ligands and exquisite ICT sensitivity, initially reported in classic Hodgkin’s lymphoma, and recently as rare events in various solid tumors, including HNSC (27). Despite these emerging 9p-related immune gene and ICT effects, attempts to dissect 9p have failed to reveal a clear candidate mediator, likely to be tissue specific, of immune response and ICT benefit. Here, we extend the 9p/IO research to whole-exome and whole-transcriptome, continuous variable dissection of somatic alterations of 9p21.3, 9p24.1, or both, including analyzing the influence of deletion depth and degree of somatic gain (at locus, band, and gene levels) in four HPV^–^ HNSC (and 10 other solid tumor) cohorts, to computationally assess their copy number and transcript contributions to immune-cold or -hot phenotypes and resistance or sensitivity to immunotherapy.

## Results

### 9p24.1 SCNA Is Associated With TME Phenotype in HPV^–^ HNSC.

Among 343 HPV^–^ HNSC patients with genomic SCNA data derived from The Cancer Genome Atlas (TCGA), copy number loss frequencies for 9p arm, 9p21.3, and 9p24.1 were 34%, 48%, and 42%, and copy number gain frequencies for 9p arm, 9p21.3, and 9p24.1 were 14%, 17%, and 22%, respectively. In this analysis, we explored band loss at the “deep” and homozygous deletion levels, as recently reported (3, 22), as well as tumors with high-level (>2.5 copies) gains (Fig. 1*B* and *SI Appendix*, Figs. S1 and S2 and Dataset S1). The results confirm the high rates of 9p21.3 band loss (19) but also reveal data on the high rates of deep deletions in this band specifically (i.e., less frequent in 9p24.1), controlling for tumor ploidy (more than 50% log2 transformed copy number loss). We first studied the association between SCNA (gain or loss) events of each chromosomal region along the genome and the immune score or CD8 T-cell level by using multivariable logistic regression after controlling for overall SCNA level (Fig. 1 *C* and *D*). In brief, the cytotoxic immune score was based on the RNA expression of the cytotoxic markers *GZMH*, *PRF1*, *CD3E*, *CD247*, *CD2*, *GZMK*, and *NKG7* (2). CD8 T-cell levels were evaluated by Microenvironment Cell Populations–counter (28), a deconvolutional method based on the normalized log2-transformed gene expression matrix to infer the absolute abundance scores for CD8 T-cell level, and the results were validated by several other deconvolutional methods and CD8 expressions: quanTIseq (29), CIBERSORT (30), xCELL (31), and *CD8A* and *CD8B* RNA expression. We used *z*-score (Fig. 1*D*) in multivariable analysis for each chromosomal region to represent the association between immune infiltrates and SCNAs in the corresponding chromosomal regions (after controlling for the SCNA level), with a positive and negative *z*-score indicating, respectively, positive and negative associations of SCNA events with immune infiltrates. The results for CD8 T-cell level associations were highly consistent with six different methods and markers mentioned above (Dataset S2). In addition to negative associations of 9p loss (notably strong for 9p24.1) with immune score, with β (β-coefficient) = –1.23, *q*-value (false discovery rate [FDR] adjusted *P*) = 9.3E-4, 9p arm gain (peak at 9p24.1 shown by arrows in Fig. 1*D*, *Right*) had a similarly strong positive association with immune score (β = 1.84, q = 6.8E-4) and CD8 T-cell levels (β = 1.60, q = 1.82E-3), (Datasets S1 and S2).

To assess the relative contributions of 9p21.3 and 9p24.1 loss to immune-cold TMEs, we applied a multivariable logistic model (after controlling for both 9p arm loss and SCNA level) to predict cytotoxic immune score or CD8 T-cell levels, again using focal events (excluding arm-level events). Consistent with previous studies and analyses above, both 9p arm loss and SCNA levels were significant predictors of low immune score (9p arm loss: β = –1.60, *q* = 5.7E-5; SCNA level: β = –0.62, *q* = 1.5E-4) and CD8 T-cell level (9p arm loss: β = –1.67, *q* = 2.6E-5; SCNA level: β = –0.57, *q* = 4.5E-4) (Dataset S3). Although limited by the small sample size (*n* = 20), analysis of the 9p24.1, but not 9p21.3, loss subgroup showed a trend for the prediction of low immune score (9p24.1: β = –0.96, *q* = 0.10; 9p21.3: β = –0.39, *q* = 0.41) and CD8 T-cell level (9p24.1: β = –1.00, *q* = 0.11; 9p21.3: β = –0.35, *q* = 0.46). The significance of 9p24.1 gain was observed when we applied continuous instead of categorical SCNA values for the association (CD8 T-cell: β = 0.38, *q* = 0.07; immune score: β = 0.40, *q* = 0.06). Size effect (variable importance) analysis showed that 9p loss could explain 42% of the variance for CD8 T-cell level and 40% for immune score (Dataset S3).

To better understand SCNA gene dosage effects of 9p24.1 and 9p21.3, we examined the correlation between CD8 T-cell and SCNA levels as a continuous variable (thus including both losses and gains) for these two bands. We calculated these correlations both including all samples and after excluding the samples with no gain or loss. 9p and 9p24.1 SCNAs showed a positive correlation with CD8 T-cell level (Spearman’s rho = 0.38, *P* = 2.6E-7; and rho = 0.32, *P* = 0.04, respectively) after removal of samples with no gain or loss (Fig. 1*C* and Dataset S4). Similar results were found when we tested the full 9p24 locus (which includes the three [9p24.1, 9p24.2, and 9p24.3] bands), where 9p24 SCNA showed a positive correlation with CD8 T-cell level (Spearman’s rho = 0.34, *P* = 0.03 after removal of samples with no gain or loss, Dataset S4). In contrast, none of the correlations between the 9p21 locus or 9p21 bands (9p21.1, 9p21.2, and 9p21.3) and CD8 T-cell level showed statistical significance (Spearman’s rho = –0.07, *P* = 0.62 for 9p21.3; and rho = 0.15, *P* = 0.42 for 9p21, Dataset S4). Accordingly, we also found a positive correlation between CD8 T-cell level and *JAK2*-*CD274* (located on 9p24.1) SCNA (Spearman’s rho = 0.37, *P* = 0.04), but not for *CDKN2A*-*MTAP* SCNA (located on 9p21.3), Spearman’s rho = –0.13, *P* = 0.15 for *CDKN2A*-*MTAP* (Fig. 1*C*).

Next, we assessed whether deep loss would have a greater reduction in CD8 T-cell levels than shallow loss (see *SI Appendix*, *SI Methods* for definitions of shallow loss and deep loss). To examine this question, we split the 9p arm loss, 9p21.3 loss, and 9p24.1 loss by different depths of deletion. After we applied different deconvolutional methods as indicated above, CD8 T-cell levels were statistically lower in any loss group (shallow loss or deep loss) when compared with no loss (or wild-type) group (*SI Appendix*, Figs. S1 and S2). No significant differences were found between shallow loss and deep loss in all three region analyses. Importantly, and consistent with Han et al. (3), we also found no significant differences in CD8 T-cell levels between 9p21 loss of heterozygosity and 9p21 homozygous deletion by using the recently reported method and by using an independent computational method (*SI Appendix*, Fig. S1). Finally, we note that there is a highly significant co-occurrence of 9p21.3 deletion (arm or focal) and 9p24.1 deletion (arm or focal) (*P*=1.82E-57), and focal deletion of 9p21.3 and 9p24.1 (*P*=2.03E-07), suggesting that the effects seen for 9p21.3 loss (e.g., on PD-L1 expression) are likely due to simultaneous co-deletion of 9p24.1.

### 9p24.1 SCNA Associations With TME Phenotype in Independent HPV^–^ HNSC Validation Cohort.

To validate our findings in an independent patient cohort, we performed similar analyses on the HPV^–^ HNSC cohort from the Clinical Proteomic Tumor Analysis Consortium (CPTAC) (32). After we adjusted the SCNA by purity and ploidy as in TCGA, 108 HPV^–^ HNSC patients were available for the analysis. Among them, 20% had 9p loss and 8% had 9p24.1 focal loss. We examined the correlation between CD8 T-cell level (and immune score) and SCNAs for 9p24 and 9p24.1 considered as continuous variables (including both losses and gains). There was a positive 9p24 trend for the correlation with CD8 T-cell level (*SI Appendix*, Fig. S3*A*) and immune score (*SI Appendix*, Fig. S3*B*; Spearman’s rho = 0.58, *P* = 0.06 for immune score). Similar positive trends were observed for 9p24.1 but not for 9p24.2 and 9p24.3 (*SI Appendix*, Fig. S3), albeit limited by the small sample size of this dataset (*n* = 10). Taken together, the results showed that an important SCNA contributor to the association between 9p and TME (CD8 T-cell level and immune score) was 9p24.1; our results attribute a less significant effect of 9p21.3 to TME (Spearman’s rho = 0.44, *P* = 0.16 for CD8 T-cell level).

### Tissue-Specific 9p21.3 and 9p24.1 Dosage Effects on TME Phenotype in Different Solid Cancers.

**Fig. 2.**
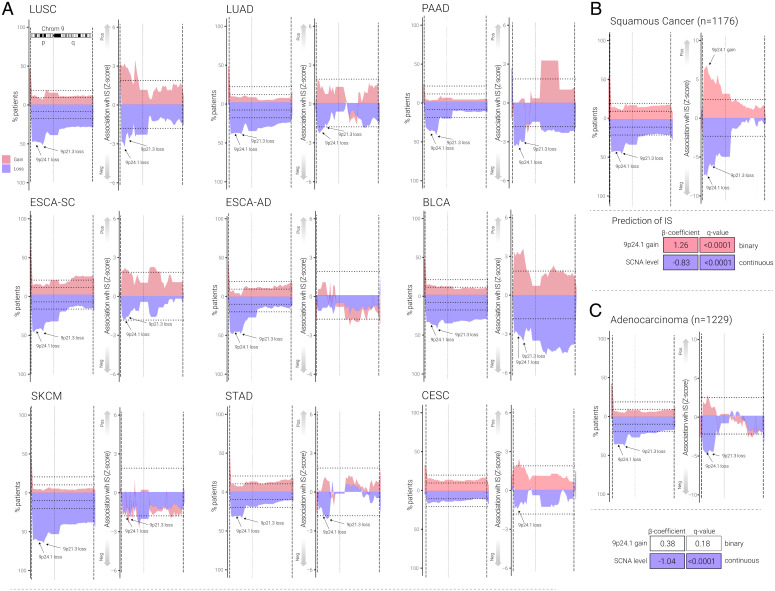
Association between 9p and immune infiltrates on other solid tumor types. (*A*) Area plot represents the percentage of patients and association with immune score for other cancer types (LUSC, LUAD, PAAD, ESCA-squamous [ESCA-SC], ESCA-adenocarcinoma [ESCA-AD], BLCA, SKCM, STAD, and CESC) as indicated. For each cancer type, *Left* panel represents the percentage of patients having SCNA on chromosome 9, and the p and q arms are split by the light vertical dotted line. The horizontal dotted line represents 10% and 20% of the patients. *Right* panel represents the area plot showing the association (*z*-score) between gains (red) and losses (blue) and immune score on chromosome 9 in multivariable logistic regression model (e.g., immune score ∼9p loss + SCNA level), and black text with arrow marks the location of 9p24.1 and 9p21.3. The p and q arms are split by the light vertical dotted line. The horizontal dotted line represents *P* = 0.05. Positive *z*-score indicates the SCNA is positively associated with the immune score, and negative *z*-score indicates the SCNA is negatively associated with the immune score. The text with black arrow indicates the location of 9p21.3 and 9p24.1. Red indicates gain and blue indicates loss. (*B*) Area plot represents the percentage of patients and association with immune score on chromosome 9 for squamous cancer types (HPV^–^ HNSC, LUSC, ESCA-SC and CESC), black text with arrow marks the location of 9p24.1 and 9p21.3. *Left* panel represents the percentage of patients having SCNA on chromosome 9, and the p and q arms are split by the light vertical dotted line. The horizontal dotted line represents 10% and 20% of the patients. *Right* panel represents the area plot showing the association (*z*-score) between gains (red) and losses (blue) and immune score on chromosome 9 in multivariable logistic regression model (e.g., immune score ∼9p loss + SCNA level), and black text with arrow marks the location of 9p24.1 and 9p21.3. The p and q arms are split by the light vertical dotted line. The horizontal dotted line represents *P* = 0.05. Positive *z*-score indicates the SCNA is positively associated with the immune score, and negative *z*-score indicates the SCNA is negatively associated with the immune score. The text with black arrow indicates the location of 9p21.3 and 9p24.1. *Bottom* panel represents the beta-coefficients and *q*-value from a multivariable logistic regression between immune score and sharp peak at 9p24.1 focal gain in squamous cell cancers. (*C*) Area plot represents the association (*z*-score) between gains (red) and losses (blue) and immune score on chromosome 9 for adenocarcinomas (LUAD, PAAD, STAD, and ESCA-AD), and black text with arrow marks the location of 9p24.1 and 9p21.3. *Left* panel represents the percentage of patients having SCNA on chromosome 9, and the p and q arms are split by the light vertical dotted line. The horizontal dotted line represents 10% and 20% of the patients. *Right* panel represents the area plot showing the association (*z*-score) between gains (red) and losses (blue) and immune score on chromosome 9 in multivariable logistic regression model (e.g., immune score ∼9p loss + SCNA level), and black text with arrow marks the location of 9p24.1 and 9p21.3. The p and q arms are split by the light vertical dotted line. The horizontal dotted line represents *P* = 0.05. Positive *z*-score indicates the SCNA is positively associated with the immune score, and negative *z*-score indicates the SCNA is negatively associated with the immune score. The text with black arrow indicates the location of 9p21.3 and 9p24.1. Red indicates gain and blue indicates loss. *Bottom* panel represents the beta-coefficients and *q*-value from a multivariable logistic regression between immune score and 9p24.1 focal gain in adenocarcinoma.

To examine 9p21.3 and 9p24.1 SCNA frequency and immune marker patterns across different solid tumor types, we performed analyses similar to those above for HPV^–^ HNSC on data derived from TCGA for 10 other cancer types, nine with 9p24.1 loss frequencies of >15%, ranging from 17% for cervical squamous cancer (CESC) to 64% for skin cutaneous melanoma (SKCM) shown in Fig. 2 and Dataset S1. We also included colorectal adenocarcinoma (COADREAD) as a control example of a common solid tumor type with infrequent (10%) 9p loss (Dataset S1). Using similar methods to above (in Fig. 1 *B*–*D*), we found that in the nine tumors with frequent 9p24.1 loss (>15%), this loss event was statistically significantly associated with lower cytotoxic immune score and CD8 T-cell levels (Dataset S1). When we examined the two 9p bands individually, we found that 9p24.1 loss was associated with lower immune scores in lung squamous cell carcinoma (LUSC), lung adenocarcinoma (LUAD), pancreatic adenocarcinoma (PAAD), bladder cancer (BLCA), esophageal cancer (ESCA)-squamous but not ESCA-adenocarcinoma, CESC, SKCM, COADREAD or STAD (stomach adenocarcinoma) (Dataset S1). In contrast, 9p21.3 loss was associated with lower immune scores in LUSC, PAAD, BLCA, and STAD only. Interestingly, CESC showed a statistically significant association with 9p24.1 gain but no significant association with 9p21.3 loss, 9p21.3 gain, or 9p24.1 loss. The key finding from these tumor-specific 9p band loss or gain associations was that 9p24.1 gain was associated with higher immune scores in all five squamous cancers: HPV^–^ HNSC, LUSC, BLCA, ESCA-squamous, and CESC (Fig. 2 and Dataset S1). Pan-cancer analysis of 9p24.1 gain and immune score (*SI Appendix*, Fig. S5) in 36 different cancer types showed that only five cancer types showed significant associations between immune score and 9p24.1 gain, and they are all squamous cancer types (*SI Appendix*, Fig. S5 and Dataset S1). These results show tissue specificity for 9p-related SCNA/TME associations, with a broad association of overall SCNA level and 9p loss with immune-cold phenotypes in multiple cancers, a more prominent immune-cold effect of 9p21.3 loss in PAAD, and an association of 9p24.1 gain with immune-hot phenotypes restricted to squamous tumors including BLCA (33), and for LUSC, the latter of which was recently shown to cluster closely in SCNA profiles with HPV^–^ HNSC (33). Indeed, the 9p24.1 gain/immune hot association was readily apparent when we grouped squamous cell cancer histologies (see arrows in Fig. 2*B*) but not evident in an analysis of adenocarcinomas combined (Fig. 2*C* and *SI Appendix*, Fig. S4 and Datasets S3–S5).

### Whole Transcriptome Sequencing Reveals 9p-Dosage, TME Correlates in HPV^–^ HNSC.

**Fig. 3.**
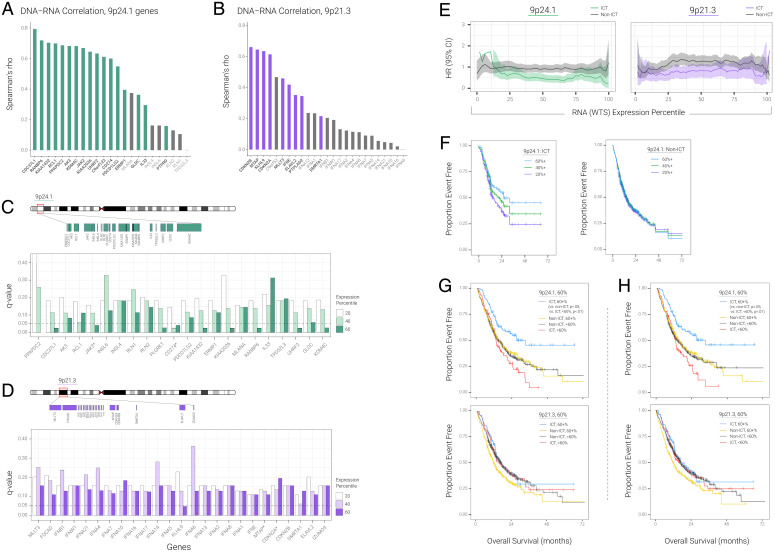
9p24.1 transcriptome predicts better survival in HPV^–^ HNSC. (*A*) DNA–RNA Spearman correlation for genes on 9p24.1 (TCGA HPV^–^ HNSC); gray color indicates low-expression genes. (*B*) DNA–RNA Spearman correlation for genes on 9p21.3 (TCGA HPV^–^ HNSC); gray color indicates low-expression genes. (*C*) Survival difference between ICT and non-ICT by genes on 9p24.1. The histogram represents the *q*-value for different percentile. For example, for gene *CD274*, white histogram represents the survival difference between ICT patients and non-ICT patients using more than 20th percentile expression of *CD274*. Light green histogram represents survival difference between ICT patients and non-ICT patients using more than 40th percentile expression of *CD274*. Green histogram represents survival difference between ICT patients and non-ICT patients using more than 60th percentile expression of *CD274*. Dashed line represents the significance of *q* = 0.05. Only genes with HR < 1 and *q* < 0.05 would pass the final filter. All the genes are sorted by genome coordinates. (*D*) Survival difference between ICT and non-ICT by genes on 9p21.3. For example, for gene *CDKN2A*, white histogram represents the survival difference between ICT patients and non-ICT patients using more than 20th percentile expression of *CDKN2A*. Light purple histogram represents survival difference between ICT patients and non-ICT patients using more than 40th percentile expression of *CDKN2A*. Purple histogram represents survival difference between ICT patients and non-ICT patients using more than 60th percentile expression of *CDKN2A*. Dashed line represents the significance of *q* = 0.05. Only genes with HR < 1 and *q* < 0.05 would pass the final filter. All the genes are sorted by genome coordinates. (*E*) HR for ICT-treated vs. non-ICT-treated groups at each of the different RNA expression percentile thresholds for 9p24.1 and 9p21.3. (*F*) Kaplan–Meier curves for overall survival, measured from specimen collection date through last as a function of cumulative RNA expression based on cohorts for patients above versus below the 20th, 40th, and 60th percentile for 9p24.1 treated with and without ICT. (*G*) Kaplan–Meier curves for overall survival, measured from specimen collection date through last as a function of cumulative RNA expression based on cohorts for patients above versus below the 60th percentile for 9p24.1 (9p21.3) treated with and without ICT. (*H*) Kaplan–Meier curves for overall survival as in (*F*) but in the PD-L1 IHC-positive subgroup only.

We evaluated the correlation of 9p24.1 and 9p21.3 between DNA and RNA in HPV^–^ HNSC in two independent cohorts (Fig. 3 *A* and *B* and *SI Appendix*, Fig. S6*A*), as a transition from the above Figs. 1 and 2, which analyzed the association between DNA and TME, to the following RNA–TME associations shown in Fig. 3 *C*–*H*. To understand the association between SCNA and gene expression, we applied DNA–RNA Spearman’s correlation in the TCGA HPV^–^ HNSC cohort. After we removed low-expression genes, the median Spearman’s rho coefficient for 9p24.1 was 0.63 and the median Spearman’s rho coefficient for 9p21.3 was 0.46. In addition to Fig. 3 *A* and *B*, which show DNA–RNA correlation based on Spearman’s correlation, we included similar DNA–RNA correlations by using Pearson’s correlation for 9p24.1 and 9p21.3 (*SI Appendix*, Fig. S6 *A* and *B*, respectively). No significant differences were found for the DNA–RNA correlation between 9p24.1 and 9p21.3 (*P* = 0.22). Importantly, all patients in the same region show similar SCNA gain and loss trends (median variance = 0.65 for 9p24.1 and 0.61 for 9p21.3; *SI Appendix*, Fig. S6*C*). Next, we evaluated the correlation of RNA expression from whole transcriptome sequencing (WTS) to DNA copy number from whole exome sequencing (WES) across 9p band and gene levels in an independent HPV^–^ HNSC cohort of 1,746 patients (real world cohort [RWC]). Consistent with our TCGA analyses (e.g., in Fig. 3 *A* and *B*), 9p24.1 gene dosage derived from WTS tracked closely with copy number determined by WES, with Spearman’s rho coefficient of 0.746 (*P* < 1.0E-4). We then focused our analyses on computing the WTS associations of 9p24.1 or 9p21.3 with CD8 T-cell levels and found that the 9p24.1 transcript correlate, *JAK2*-*CD274*, was more highly correlated with *CD8A/B* levels (rho = 0.61/0.55, *P* < 1.0E-4) than the 9p21.3 correlate, *MTAP*-*CDKN2A*, as recently reported (3) for *CD8A/B* (rho = 0.21/0.17). These results are consistent with TCGA findings (Dataset S6) supporting the hypothesis that 9p24.1 plays a larger role in HPV^–^ HNSC TME activation than 9p21.3.

### 9p24.1 Transcriptomic Variation Predicts Anti-PD-1 ICT Benefit in HPV^–^ HNSC.

Based on the strong, consistent (from three independent cohorts above) 9p24.1 association with CD8 T-cell TME activation in WES and WTS datasets, we hypothesized that 9p24.1 transcript level could represent a biomarker that, in addition to (and potentially in lieu of) PD-L1 protein expression, could more accurately predict clinical benefit from PD-1-targeted agents in HPV^–^ HNSC. To test this hypothesis, we analyzed 9p24.1 gene dosage associations from WTS profiles with patient survival after ICT immunotherapy in a de-identified, RWC dataset of 894 HPV^–^ HNSC patients with recurrent/metastatic disease: 208 patients received first- or second-line anti-PD-1 checkpoint therapy (pembrolizumab, nivolumab), and 694 patients had been treated with chemotherapy (with no prior or subsequent ICT). In our initial approach, focused on the ICT-treated group only, we evaluated every gene in the 9p21.3 and 9p24.1 bands individually to determine whether expression levels of each gene singly could stratify patient survival after ICT. When we accounted for false discovery, of the 25 genes at 9p21.3, only one gene, at one percentile threshold (KLHL9 at the 60th percentile threshold) was significantly associated with survival (*q*-value < 0.05, hazard ratio [HR] < 1), whereas 9 of the 22 genes at 9p24.1 were statistically significantly associated with ICT survival at the 60th percentile (*q* < 0.05, HR < 1) (Fig. 3 *C* and *D*). The difference between 9p24.1 (9/22) and 9p21.3 (1/25) genes associated with ICT survival at the 60th percentile was significant (*P* = 0.005). Importantly, many more 9p24.1 gene percentiles had statistically significant *P* values that did not survive false discovery correction (*AK3*, *ERMP1*, *GLDC*, *INSL6*, *PDCD1LG2*, *RCL1*, *RLN1*, and *RLN2*); we did not see similar survival patterns in 9p21.3 after false discovery correction, although we did observe *P* < 0.05 in each of *DMRTA1*, *ELAVL2*, *FOCAD*, *IFNA1*, *IFNA13*, *IFNA16*, *IFNA17*, *IFNA2*, *IFNA6*, *IFNA7*, *IFNE*, and *MTAP*. These data show an inherent 9p band-level difference in shaping immune response and suggest that 9p24.1 is a relative hotbed of immune regulatory genes.

We next investigated the contributions of *JAK2* or *CD274* when analyzed individually or combined. Given the high correlation and colinearity of *JAK2* and *CD274*, we plotted HR metrics from a Cox proportional hazards model for survival after ICT versus chemotherapy according to percentile expression of each gene alone or combined. The maximum HR differences between overexpressors and underexpressors occurred at the 66th percentile for *JAK2*-*CD274* (Fig. 3*C*; the peak thresholds for *CD274* alone and *JAK2* alone were 44 and 70, respectively; see * in *SI Appendix*, Fig. S7*A*). We then defined a null hypothesis that the HR separations—with window width values as follows: between the expression levels of *CD274* (34th–62nd percentiles) or *JAK2* (66th–72nd percentiles) and *JAK2*+*CD274* (44th–70th percentiles)—are the same across all percentiles. The previous values are ICT-treated relative to non-ICT-treated with expression exceeding the optimal threshold. With *P* = 3.0E-3, we were able to reject the null hypothesis, indicating that not only was the *JAK2*-*CD274* peak HR difference greater than those for *CD274* and *JAK2* expressions alone, but the window width signature patterns by expression percentile were different, and the combination of the two genes together provides ICT predictive information missed by either gene alone. At this optimal threshold, patients treated with chemotherapy had the corresponding HRs of 0.9, 1.1, and 1.0, respectively, showing that the survival difference is dependent on, and specific for, administration of anti-PD-1 ICT. These findings are consistent with our earlier targeted sequencing study, where we observed a *JAK2*-*CD274* codeletion association with ICT resistance that was much stronger than either gene deletion alone (2). A Fisher’s exact test checked whether we could expect the same signal shift (ICT better than non-ICT in overexpressors, opposite in underexpressors) in the same locations along the *x*-axis. Kaplan–Meier survival analyses at the optimal points provided an independent assessment and independent *P* value correction (*SI Appendix*, Fig. S7*B*). For each percentile, we assessed whether the overexpressor cohort showed the same survival benefit to ICT or standard therapy. When *P* < 0.05, we rejected the null hypothesis that the survival benefit is the same for the ICT-treated and non-ICT-treated cohorts. Among *JAK2*-*CD274* overexpressors at the 66th percentile, the comparisons were significant at *P* < 0.0005 and *q* = 0.019, an FDR adjustment of a log-rank *P* value (see *SI Appendix*, *SI Methods*). At a standard 5% alpha on the FDR, we expected that 5% of the evaluated percentiles that are called “significant” would actually be null (i.e., no survival difference). The observed *q* < 0.05 indicates that the *CD274*+*JAK2* signature at the 66th percentile is associated with a survival benefit in ICT-treated (relative to non-ICT-treated) patients (*SI Appendix*, Fig. S7*B*). ICT-treated patients with expression less than the defined cutoff had lower survival compared to those treated with chemotherapy, although only *CD274* less than the 44th percentile achieved statistical significance (HR = 1.399; 95% CI, 1.046–1.872; log-rank *P* = 0.023).

We next computed HRs for survival as a function of 9p21.3 represented by the *CDKN2A*-*MTAP* transcript, as assessed in recent reports (3, 22) and for 9p24.1 (represented by *JAK2*-*CD274*) dosage percentiles in a continuous variable analysis, using the lowest percentile as the reference group. We found decreasing HRs with increasing *JAK2*-*CD274* transcript expression dosage in ICT-treated patients (with the curves crossing HR = 1 at 20th expression percentile) but not in chemotherapy-treated patients. HRs remained relatively unchanged with increasing *CDKN2A*-*MTAP* expression dosage (Fig. 3*E*). Analyses of median overall survivals or relative risks of death showed similar patterns (*SI Appendix*, Fig. S8 *A* and *B* and Dataset S7). These results support the role of 9p24.1 (but not 9p21.3) transcript down-regulation or up-regulation (and specifically *JAK2*-*CD274*) as a predictive biomarker of ICT resistance or sensitivity, respectively, in HPV^–^ HNSC. Kaplan–Meier survival plots for the 20th, 40th, and 60th RNA percentiles for 9p24.1 treated with anti-PD-1 therapy revealed superior survival of the top (vs. bottom) 40% expression subgroup (HR = 0.58; 95% CI, 0.387–0.873; log rank *P* = 0.008; Fig. 3*F*); there were no significant differences by 9p24.1 expression percentile in the non-ICT group (HR = 1.115; CI, 0.899–1.383; Fig. 3*F*). Consistent with the above non-ICT-treated RWC data, when we applied the Cox proportional hazards regression model to predict survival rates in the non-ICT HPV^–^ HNSC patients in TCGA, most of the genes on either 9p21.3 or 9p24.1 did not show significant survival differences when we used the 20/40/60% expression percentiles (*SI Appendix*, Fig. S9).

The selective predictive effects of *JAK2*-*CD274* transcript expression are shown by the inferior Kaplan–Meier survival curve of ICT-treated patients (compared to chemotherapy-treated patients) in the <60th percentile subgroup, in sharp contrast to the superior survival of ICT monotherapy in the subgroup with the highest 40th transcriptome dosage percentile (Fig. 3*G* and Dataset S7). Similar results were observed when we used the three-gene amplicon (including *PDCD1LG2*) at 9p24.1 (maximum survival difference for *JAK2*+*CD274* is 118%; for *JAK2*+*CD274*+*PDCD1LG2* it is 124%). Notwithstanding the prominent roles observed with genes at 9p24.1, there is an apparent influence of larger deletions and 9p21.3 gene-level contribution as well, as *KLHL9* from 9p21.3 was statistically significant at the 60% threshold after false discovery correction (Fig. 3*D*). *KLHL9* expression added to the maximum overall survival difference observed for *JAK2*-*CD274* (129% for *JAK2*+*CD274*+*KLHL9* vs. 118% for *JAK2*+*CD274*). Because PD-L1 (*CD274*) immunohistochemistry (IHC) protein expression is routinely used in clinical practice to select patients for ICT, we assessed whether *JAK2*-*CD274* transcriptome dosage could further identify PD-L1-positive patients most likely to benefit from ICT or chemotherapy. Within the subgroup of 803 patients (of the total 894 RWC) with standard binary PD-L1 combined positive score protein expression ≥1, *JAK2*-*CD274* transcript levels <60th percentile identified PD-L1 IHC-positive patients with survival rates inferior to those of chemotherapy (Fig. 3*H*).

Finally, we assessed 9p21.3 and 9p24.1 expression associations with CD8 T-cell levels in an independent cohort of patients with HPV-positive HNSC (*n* = 556; *SI Appendix*, Fig. S10 *A* and *B*). HPV-positive HNSC showed lower correlation between *CD8A* and expression of *JAK2* and *CD274* (Pearson’s *r* = 0.501, *P* < 0.05 for HPV^–^ HNSC and Pearson’s *r* = 0.049 for HPV-positive). Similar results were found also for the correlation between *CD8B* and expression of *JAK2* and *CD274* (Pearson’s *r* = 0.403, *P* < 0.05 for HPV^–^ HNSC and Pearson’s *r* = 0.004 for HPV-positive; *SI Appendix*, Fig. S10*B*). Consistent results were also confirmed when we used the TCGA dataset (HPV-positive HNSC, *n* = 43); no significant association was found between 9p21.3 and 9p24.1 SCNA and immune score (*SI Appendix*, Fig. S10). Taken together, these data suggest that the association between 9p24.1 and 9p21.3 and CD8 or immune score in HNSC was mostly limited to HPV^–^ HNSC, with weaker associations in HPV-positive HNSC.

## Discussion

HPV^–^ head and neck cancer, the most common and lethal subtype of head and neck cancer with over 200,000 deaths globally per year, is characterized by extensive somatic genomic copy number alterations. Here, we demonstrated that 9p24.1 genetic dosage significantly contributed to an immune-cold or -hot phenotype (when genes are lost or gained, respectively) in HPV^–^ HNSC, in WES and WTS analyses of three independent cohorts, which in turn predicted resistance and sensitivity to standard anti-PD-1 ICT in a fourth real-world patient cohort with recurrent/metastatic disease. The contributions of 9p21.3 to immune TME activation and ICT response were less prominent or nonexistent. These data build on our previous report demonstrating that 9p somatic copy number loss in HPV^–^ HNSC was associated with immune-cold tumor microenvironments and poor survival after anti-PD-1 immunotherapy (2). There have been two subsequent solid tumor studies of 9p21.3 loss (inferred from two genes on this band, *CDKN2A* and *MTAP*) reporting that 9p21.3 loss was associated with TME or ICT outcomes in lung adenocarcinoma, bladder cancer, melanoma, and small mixed solid tumor ICT cohorts (3, 22). Both reports included too few patients (17 in each report, HPV status unclear) with HNSC to analyze separately, but these HNSC patients were included in ICT outcome analysis of mixed solid tumor cohorts. The largest study was a pan-tumor study of *CDKN2A* and *MTAP* expression as a surrogate for 9p21.3 heterozygous or homozygous deletion, which confirmed our earlier HNSC/TME findings (3), specifically showing that 9p21.3 loss in HPV^–^ HNSC was associated with immune-cold, CD8 T-cell depleted TME. Analyses here (*SI Appendix*, Fig. S1) were consistent with this latter report that showed no difference between heterozygous loss of heterozygosity and homozygous 9p21.3 deletion on TME in HPV^–^ HNSC, and the authors speculated that 9p24.1 may be co-lost with 9p21.3. A second, smaller study of 9p21.3 “deep” deletions (22) reported inferior survival trends in an analysis of mixed solid tumor cohort of 87 patients. A third recent study reported that 9p21.3 loss (as assessed by *CDKN2A*, *CDKN2B* plus *MTAP*) was associated with poor survival after anti-PD-1 monotherapy but not in ICT–chemotherapy combination treated patients with nonsquamous lung cancer (34). The ICT monotherapy findings remained, albeit less statistically significant, in a subgroup of PD-L1-positive patients.

Previous work from our group and others has demonstrated that 9p21.3 and 9p loss are among the most common focal and arm events in human cancer (19, 20). In the current report, we assessed somatic 9p band alterations as a continuous variable and demonstrated that 9p24.1 loss was associated with immune-cold, CD8 T-cell-depleted HPV^–^ HNSC, whereas 9p21.3 focal loss was not. Both 9p21.3 and 9p24.1 loss and gain frequencies were similar and frequently occurred as part of an arm-level event, which could confound previous analyses on the specific influence of regional 9p21.3 alterations on immune TME activation when such effects were indeed due to coalterations in 9p24.1. Our analyses (1) support the hypothesis that 9p24.1 is a somatic alteration key to shaping the immune TME response, probably with more modest contributions of alterations in genes located elsewhere in 9p (e.g., *KLHL9*, Fig. 3*D*) and other chromosomes (2), and justify the development of 9p-related biomarker tests, more specifically 9p24.1 (or *JAK2*-*CD274* transcriptomic correlate), as more efficient biomarker tests to select patients for ICT. Importantly, not only did we confirm and extend binary 9p loss/immune-cold/ICT resistance observations in our and other recent reports, we demonstrated 9p24.1 gain as a possible driver of an immune activation and ICT response in HPV^–^ HNSC and several other squamous cancers (Fig. 2*B*).

The mechanisms behind somatic 9p24.1 dosage effects on immune TME remain to be elucidated. PD-L1 expression is often considered a result of a downstream effect from IFN-γ signaling in the context of immune infiltration; PD-L1 loss or gain alone therefore would be unlikely to directly influence TME, even though it could determine how tumors escape after immune activation. Specific 9p24.1 alterations relevant to both TME and response or resistance to immunotherapy include the IFN-γ pathway gene *JAK2*. *JAK2* gain or loss of function somatic alterations can promote or suppress PD-L1 expression, respectively, which affects TME and ICT response. In contrast to PD-L1, one could postulate a direct, broad effect of *JAK2* alterations on TME, and a pivotal role of *JAK2* in cancer cell sensitivity to IFN-γ, impaired T-cell sensitivity, and evasion (35), by modulating the degree of PD-L1 expression and antigen presentation upon IFN-γ release (36), further augmenting or dampening immune response. As an example, in triple-negative breast cancer cell lines with 9p24.1 gain, PD-L1 expression was markedly inducible by low-dose IFN-γ in a copy-number dependent manner, mimicking an in situ inflammatory response (37). An enhanced, PD-L1-enriched, inflammatory response could explain the immune-hot phenotype observed in HPV^–^ HNSC with 9p24.1 gain in our study, consistent with a recent report of the CD8+ T-cell inflamed phenotype in HNSC samples enriched by *CD274*, *PDCD1LG2*, *JAK2*, and *KDM4C* at 9p24.1 amplification (38). The 9p24.1 gain/immune-hot association seems to be tissue specific and prominently featured in squamous cell histologies, driven primarily by HPV^–^ HNSC and squamous cell carcinomas of the lung (Fig. 2), which has been reported to track experimentally and computationally with HPV^–^ HNSC in pan-cancer genomic SCNA association studies (33, 39), possibly reflecting shared coevolution of immune evasion and neoplastic invasion (1, 21, 40–42). This highlights the importance of determining mechanisms of 9p band somatic alteration–related immune modulation in different tumor types, especially if these genomic features are to be used as biomarker tests to guide precision ICT. In PAAD, for example, we found that 9p21.3 loss was the prominent driver of low immune score/CD8 T cells, in accordance with recent evidence in pancreatic cancer mouse models (43).

The strong associations between 9p24.1 gene dosage and immune TME open the opportunity for biomarker development to guide ICT in HPV^–^ HNSC and other tumor types. In support of this application, we demonstrated that high expression levels of nearly half of the genes in 9p24.1 were associated with ICT benefit, whereas only one gene in 9p21.3 was (Fig. 3 *C* and *D*), prompting reexamination of the role of 9p21.3 as a predictive marker in HPV^–^ HNSC. Two recent studies correlated *CDKN2A*/*MTAP* loss as an ICT resistance marker in several pan cancers but were limited by the small number of HPV^–^ HNSC patients (3, 22) and could not evaluate the contributions and interactions of other chromosomal sites or genes to this observation. Our large RWC dataset analyses pointed to the strong survival associations of *JAK2*-*CD274* dosage, with anti-PD-1 monotherapy producing a threefold increase in median survival at the 60th percentile expression, and above threshold.

Although we found consistent, statistically significant associations of *CD274* and *JAK2* with anti-PD-1 response in our HPV^–^ HNSC RWC, our data strongly support the IO importance of several other genes at 9p24.1, including *RANBP6* and *KDM4C*. Amplification of the latter gene was associated with TME hot in a recent HNSC report (38), rather than *CDKN2A*-*MTAP*, limited to ICT-treated (not chemotherapy-treated) patients. Notably, the highly selective predictive effects of *JAK2*-*CD274* transcript levels were bidirectional and could identify patients who had ICT outcomes inferior to those of chemotherapy in the low-expression groups, in resonance with the somatic alteration immune cold/hot phenotype associations. These 9p24.1 effects were evident even within the group with PD-L1 combined positive score ≥1 (Fig. 3*H*), suggesting this to be a biomarker test to refine the predictive value of PD-L1 alone to assess resistance and sensitivity to ICT in HPV^–^ HNSC. Approximately 80% of patients with HNSC have PD-L1 IHC-positive tumors; however, the majority of these patients do not benefit from ICT. Here we show that the lowest 60 percentiles of patients by *JAK2*-*CD274* expression have greater clinical benefit when treated with chemotherapy than with ICT. Finally, since 9p arm-level loss is the strongest predictor of TME in HPV^–^ HNSC (Dataset S1) and other tumor types, there are probably other genes on 9p that cooperate with genes on 9p24.1 to promote immune-cold TME.

In summary, this report provides multiomic evidence from four cohorts that the spectrum of immune TME alterations from cold to hot in HPV^–^ HNSC are highly influenced by somatic alterations in 9p24.1 dosage in HPV^–^ HNSC, with 9p21.3 genes playing a secondary role. The wide application and low response rates of ICT make it imperative to develop biology-driven, accurate biomarker tests that clinicians can use to help predict and guide therapy (or at least be complementary to other measures), thus sparing toxic, costly, and potentially nonefficacious treatment. When the complexity of the immune system is taken into consideration, it becomes increasingly evident that we will have to integrate, through comprehensive multiomic immune evaluations, both genomic and nongenomic biomarkers in the predictive tools for ICT response and resistance, if all aspects of the cancer–immunity cycle are to be encompassed in decision-making algorithms. Within this context, *JAK2*-*CD274* expression may need to be incorporated, in addition to standard binary PD-L1 immunohistochemistry, into anti-PD-1 ICT-based strategies to maximize precision treatment, not only for HPV^–^ HNSC but potentially for other (squamous) solid tumors in which 9p24.1 dosage shapes TME.

## Methods

### Datasets in TCGA.

Arm and gene-level SCNA, gene expression, HPV status, and clinical parameters for HPV^–^ HNSC, LUAD, LUSC, PAAD, BLCA, SKCM, ESCA-squamous, ESCA-adenocarcinoma, STAD, CESC, and COADREAD were derived from the TCGA dataset. Copy number (given as log2 copy number ratios) data for the 11 cancers above in the TCGA cohort were derived from Affymetrix SNP 6.0 arrays and obtained from GISTIC2 analysis (level 4). Gene expression files were obtained from RSEM analysis (level 3). All TCGA-related files can be downloaded from Broad GDAC Firehose (v20160128). See *SI Appendix* for additional method details, including SCNA classification, variable importance analysis, CD8 T-cell deconvolution, associations between CD8 T-cell and SCNA levels, and associations between immune score and SCNA levels.

### Datasets in CPTAC.

For CPTAC3 HNSC (32), log2 ratio gene level and segment SCNA as well as gene expression and clinical parameters used in the analysis can be obtained from LinkedOmics (http://www.linkedomics.org). The HPV status was filtered by clinical parameters “HPV inference.” GISTIC2 (44) was applied to generate the 9p arm and 9p24.1, 9p24.2, 9p24.3 focal-level SCNA with default parameters. 9p24 and 9p21 SCNA was evaluated based on the median of genes located on 9p24.1, 9p24.2, and 9p24.3; and 9p21.1, 9p21.2, and 9p21.3, respectively.

### Dataset From the RWC.

A total of 1,746 HPV^–^ HNSC cases with WTS and WES data and a total of 894 HPV^–^ HNSC cases with WTS and outcome data available were used for this study. Immune hot was defined as WTS TPM for *CD8A* and *CD8B* both being greater than the median TPM values for *CD8A* and *CD8B*, respectively. See *SI Appendix* and Datasets for additional analyses including DNA copy number estimation, correlation analysis between WTS and WES, and RWC analysis.

## Supplementary Material

Supplementary File

Supplementary File

Supplementary File

Supplementary File

Supplementary File

Supplementary File

Supplementary File

Supplementary File

## Data Availability

All study data are included in the article and/or supporting information.
