# Supplementary material for: Somatic 9p24.1 alterations in HPV– head and neck squamous cancer dictate immune microenvironment and anti-PD-1 checkpoint inhibitor activity
Source: Proc Natl Acad Sci U S A. 2022 Nov 17;119(47):e2213835119. doi: 10.1073/pnas.2213835119 (PMC9704728; doi:10.1073/pnas.2213835119)
Supplement: Supplementary File [file pnas.2213835119.sapp.pdf]

PNAS

Supporting Information for

Somatic 9p24.1 alterations in HPV⁻ head and neck squamous cancer dictate immune microenvironment and anti-PD-1 checkpoint-inhibitor activity

Xin Zhao[1#], Ezra E.W. Cohen[2,3], William N. William Jr[4,5], Joy J. Bianchi[1], Jim P. Abraham[6], Daniel Magee[6], David B. Spetzler[6], J. Silvio Gutkind[2,7], Ludmil B. Alexandrov[2,8,9], Webster K. Cavenee[2,3,10#], Scott M. Lippman[2,3,4$#], Teresa Davoli[1$]

[1]Department of Biochemistry and Molecular Pharmacology, Institute for Systems Genetics, New York University Langone Health, New York, NY 10016; [2]Moores Cancer Center, University of California San Diego, La Jolla, CA 92037; [3]Department of Medicine, University of California San Diego, La Jolla, CA 92037; [4]Thoracic / Head and Neck Medical Oncology, The University of Texas MD Anderson Cancer Center, Houston, TX 77030; [5]Hospital BP, a Beneficência Portuguesa de São Paulo, 01323-001 São Paulo, Brazil; [6]Research and Development, Caris Life Sciences, Irving, TX 75039; [7]Department of Pharmacology, University of California San Diego, La Jolla, CA 92037; [8]Department of Cellular and Molecular Medicine, University of California San Diego, La Jolla, CA 92037; [9]Department of Bioengineering, University of California San Diego, La Jolla, CA 92037; [10]Ludwig Institute for Cancer Research, University of California San Diego, La Jolla, CA 92037

Contributed by Webster K. Cavenee

[$]These senior authors contributed equally to this work

[#]Corresponding Authors:
Xin Zhao, PhD
Department of Biochemistry and Molecular Pharmacology
Institute for Systems Genetics
NYU Langone Health
New York, NY 10016
Phone: 212-263-8081;
Email: xin.zhao@nyulangone.org, teresa.davoli@nyulangone.org

Webster K. Cavenee, PhD
Distinguished Professor of Medicine Emeritus
University of California San Diego
9500 Gilman Drive #0660
La Jolla, CA 92093-0660
Phone: 858-793-0864
E-mail: wcavenee@health.ucsd.edu

Scott M. Lippman, MD
Distinguished Professor of Medicine

Moores Cancer Center
University of California San Diego
3855 Health Sciences Drive
La Jolla, CA 92093
Phone: 858-822-1222
Fax: 858-822-0207
email: slippman@ucsd.edu

This PDF file includes:

Supporting text - Methods
Figures/Legends S1 to S10
Titles for Datasets S1 to S7
SI References

Other supporting materials for this manuscript include the following:

Datasets S1 to S7

Methods – Extended Description

SCNA classification in TCGA and CPTAC.
The copy number for each chromosomal region (given as log2 copy number ratios) were adjusted by tumor purity and ploidy derived from previously reported ABSOLUTE (1) methods. Arm-level copy-number related purity and ploidy was downloaded from TCGA Firehose Legacy (https://gdac.broadinstitute.org). For CPTAC, purity was determined from column "tumor pathology review" and ploidy was generated based on the mean ploidy of HPV⁻ HNSC in TCGA. Then for each patient, purity α, ploidy τ, integer copy number q(x) together with arm-level copy number R(x) was applied to the SCNA adjustment:

$$R'(x) = \frac{q(x)}{\tau} = \frac{\alpha * \tau * R(x) + 2(1-\alpha) * R(x) - 2(1-\alpha)}{\alpha * \tau}$$

After purity adjustment, we considered a log2-transformed copy-number ratio >0.3 as a gain event and < (-0.3) as a loss event. To distinguish between arm and focal-level events, we considered a threshold of ≥70% (default value in GISTIC2) of arm length (given in units of the fraction of chromosome arm) to identify the arm-level events (as defined in Davoli et al., 2017 (2)) while all the others were considered as focal-level events. The arm level, arm or focal level and focal level data were generated based on GISTIC2 output. In a more detailed way, the arm level SCNA was derived from "broad_values_by_arm.txt". The arm or focal level SCNA was evaluated by the median of genes on the same region from file "all_data_by_genes.txt". For example, the SCNA for region 9p21.3 was based on the median SCNA of 25 genes located on 9p21.3. The SCNA for region 9p21 was based on the median of genes located on 9p21.1, 9p21.2 and 9p21.3. The focal region level SCNA was calculated by the median of genes on the same region from file "focal_data_by_genes.txt". For JAK2-CD274 or MTAP-CDKN2A SCNA in HPV⁻ HNSC, we first calculated the mean SCNA of JAK2 and CD274 for each patient, then we determined the gain and loss of JAK2-CD274 at 9p24.1 based on a cutoff of 0.3 (> 0.3 as gain and <(-0.3) as loss), after that we calculated the Spearman correlations between JAK2-CD274 SCNA and CD8 T-cell level. The same analysis was done for CDKN2A-MTAP at 9p21.3.

While in certain studies, tumor SCNAs are defined as homozygous or heterozygous, this holds primarily for germline SCNA. Defining whether cancer cells contain heterozygous (i.e., loss of one copy) or homozygous (i.e., loss of two copies) deletion is difficult. While the genome of normal cells is generally diploid, in most of the cancer cells from solid tumors the karyotype is highly aneuploid. For example, based on ABSOLUTE estimates (1), we found that HPV⁻ head and neck cancer contained an average of 2.6 copies per chromosome (Dataset S1), which meant each chromosome was represented by almost three copies, or triploid, instead of two as in normal cells. Thus, for cancer, instead of heterozygous and homozygous loss, we considered 'shallow' and 'deep' loss. More specifically, we considered tumors with log2 copy number ratio less than -1 as a deep loss (meaning that at least 50% of the chromosome copy number is lost), corresponding to 1 copy lost for a diploid genome, 1.5 copies for a triploid genome or 2 copies for a tetraploid genome (Dataset S1). On the other hand, we considered log2 copy-number ratio between -1 to -0.3 as shallow loss (meaning that at least 20% of the chromosome copy number was lost), corresponding to 0.4 copy loss for a diploid genome, 0.6 copy for a triploid genome or 0.8 copy loss for a tetraploid genome). We also analyzed the tumors using the 'homozygous' and 'heterozygous' definitions reported earlier, and the results were similar to our findings using the 'deep' and 'shallow' loss definitions above. Furthermore, we also determined tumors with log2

ratio more than 0.65 as high gain (meaning that at least 50% of the chromosome copy number was gained), 1 copy gain for diploid genome, 1.5 copy gains for triploid genome and 2 copy gains for a tetraploid genome; and log2 copy number ratio between 0.3 to 0.65 as shallow gain (meaning 25% of the chromosome was gained), corresponding to 0.5 copy gain for a diploid genome, 0.75 copy for a triploid genome or 1 copy gain for a tetraploid genome. Copy number neutral was classified as log2 ratio between -0.3 to 0.3.

The total SCNA level were defined as numbers of arms with gains or losses (see formula below):

$$SCNA\ level = \sum arm\ gains + \sum arm\ losses$$

Association between immune score and SCNA in TCGA and CPTAC.
The immune score (IS) was generated from cytotoxic immune cells markers: GZMH, PRF1, CD3E, CD247, CD2, GZMK and NKG7 (2). In brief, for each marker we ranked the gene expression across all patients belonging to same cancer and we summed the rank order to get a new list. After that, we ranked the new list again to generate the immune score. Next, we defined the tumors as having a low or high immune score using the bottom 35th and top 35th percentiles. For each SCNA, we used independent evaluation to analyze the association between immune score and gains or losses separately. The normalized SCNA level was also applied to the model. Then we examined the association between immune score and SCNA by applying a multivariable logistic model for gains and losses separately (below). We used z-scores to evaluate the association between immune score and SCNA. Positive and negative z-scores represented the positive or negative association between SCNA and immune score; all the p-values from the model were adjusted by false discovery rate (3).

$$IS \sim SCNA(gain) + SCNA\ level$$
$$IS \sim SCNA(loss) + SCNA\ level$$
$$IS \sim 9p21.3\ arm\ or\ focal\ loss\ level + 9p24.1\ arm\ or\ focal\ loss$$

Variable importance analysis in TCGA.
In order to understand which covariant had a more important role in regressions, we used the varImp function from the caret package (v6.0-90) to calculate the overall importance. Then we used the weighted overall importance to calculate the final size-effect.

CD8 T-cell deconvolution in TCGA and CPTAC.
We used 6 different methods or markers to evaluate the CD8 T-cell enrichment. 1) The R package MCP-counter (4), which was based on the normalized log2-transformed expression matrix to infer the absolute abundance scores for 8 immune cells (including CD8 T cells level). 2) quanTIseq (5), a method applied to the normalized RNA-seq data to estimate the relative proportions of 10 different immune cells (including CD8 T-cells level). 3) CIBERSORT, a method applied to the normalized RNA-seq data to estimate the relative proportions of 22 immune cell subpopulations (including CD8 T-cells level) using compartment-specific gene expression signatures (6). 4) xCELL, a webtool to perform cell type enrichment analysis from RSEM gene expression data for 64 immune types (including CD8 T-cells level) (7). 5) RNA expression levels for CD8A. 6) RNA expression for CD8B; previous studies had shown that RNA expression levels (CD8A or CD8B) of immune-cell markers highly correlated with CD8+ T-cell estimates based on immunofluorescence (8).

The different CD8 T-cell level between deep loss, shallow loss and no loss for 9p, 9p21 or 9p24 was calculated using the Wilcoxon rank-sum test and adjusted by the false discovery rate.

Associations between CD8 T-cell level and SCNA levels in TCGA and CPTAC.
We defined the tumors as having low or high CD8 T-cell level using the bottom 35th and top 65th percentiles. For each SCNA, we used independent evaluations to analyze the associations between CD8 T-cell level and gain or loss separately. We used z-scores to evaluate the association between CD8 T-cell level and SCNA levels. A positive z-score indicates that the SCNA is positively associated with CD8 T-cell level, a negative z-score indicates that the SCNA is negatively associated with CD8 T-cell level. All the p-values from the model were also adjusted by false discovery rate:

$$CD8\ T\ cell\ level \sim SCNA(gain) + SCNA\ level$$
$$CD8\ T\ cell\ level \sim SCNA(loss) + SCNA\ level$$
$$CD8\ T\ cell\ \ level \sim 9p\ arm\ loss\ + 9p21.3\ focal\ loss$$
$$CD8\ T\ cell\ level \sim 9p\ arm\ loss\ + 9p24.1\ focal\ loss$$
$$CD8\ T\ cell\ level \sim 9p21.3\ arm\ or\ focal\ loss\ level + 9p24.1\ arm\ or\ focal\ loss$$

DNA-RNA correlation analysis.
In order to understand the association between SCNA changes and expression for each gene, we applied both Spearman's and Pearson's correlation for DNA and RNA. Average gene expression less than 1 would be considered as low expressed gene and removed from the future analysis.

Statistical analysis for TCGA and CPTAC.
In addition to the bioinformatics approaches described above, a Spearman's or Pearson's correlation analysis was used to identify CD8 T-cell level correlations with 9p, 9p21.3 or 9p24.1 SCNAs by using continuous values. All data were analyzed in R (v4.1.1).

WES pipeline for RWE Cohort.
All the samples from the RWE Cohort were de-identified before any experiments and analyses. Genomic tumor DNA isolated from formalin-fixed paraffin-embedded (FFPE) tumor samples was micro-dissected to enrich tumor purity and subjected to NGS using the NovaSeq 6500 platform (Illumina, Inc., San Diego, CA). The FFPE specimens underwent pathology review to measure percent tumor content and tumor size; a minimum of 20% of tumor content in the area for microdissection was required to enable enrichment and extraction of tumor-specific DNA. Matched normal tissue was not sequenced. A custom-designed SureSelect XT assay was used to enrich whole exome whole-gene targets (Agilent Technologies, Santa Clara, CA). Somatic copy number alteration (SCNA) was tested by NGS and was determined by using CNVkit (9).

WTS pipeline for RWE Cohort.
FFPE specimens underwent pathology review to measure percent tumor content and tumor size; a minimum of 20% of tumor content in the area for microdissection was required to enable enrichment and extraction of tumor-specific RNA. For transcription counting, transcripts per million molecules was generated using the Salmon expression pipeline (10). RNA expression, as defined by transcripts per million (TPM) from the Salmon RNA expression pipeline (10) using the clinically validated Caris Whole Transcriptome Assay (11).

Gene level transcriptomic variation between ICT and non-ICT patients in 9p24.1 and 9p21.3.
For genes on region 9p24.1 and 9p21.3 that have both RNA expression and survival data: First, we split the patients into 3 independent groups based on different expression percentiles (>20th; >40th; and >60th percentile) . Then for each percentile, a Wilcoxon rank test was applied to test whether ICT patients would have better survival than non-ICT patients. All the p-values from the results were also adjusted by the false discovery rate (q-value). Only genes with q-value less than 0.05 and Hazard ratio (HR) less than 1.0 could pass the final filter.

9p24.1 immune hot (cold) copy number estimation for different RNA expression in RWE.
For RWE dataset: Immune hot is defined as WTS TPM (Transcripts Per Million) for CD8A and CD8B both being greater than the median TPM values for CD8A and CD8B respectively. For the RNA expression, cases were partitioned into deciles based on the median TPM of 22 genes (AK3, CD274, CDC37L1, ERMP1, GLDC, IL33, INSL4, INSL6, JAK2, KDM4C, KIAA1432, KIAA2026, MLANA, PDCD1LG2, PLGRKT, PPAPDC2, RANBP6, RCL1, RLN1, RLN2, TPD52L3, UHRF2) located on 9p24.1. Then, the mean CNVKit copies of 9p24.1 was calculated within each decile, a Wilcoxon test was applied to test the SCNA difference between immune hot and immune cold.

Real-world Cohort survival analysis.
We extracted the overall survival (OS) of each case from a repository of real-world evidence (RWE), defined as the biopsy date through last contact. Patients that did not have an observed claim data element within 100 days of the end of our RWE records were presumed to be deceased and uncensored, which was found to be 95% concordant to data obtained from National Death Index data (National Center for Health Statistics, Centers for Disease Control and Prevention). All other patients were considered censored. Patients with an observed anti-PD-1 checkpoint therapy were annotated as ICT-treated, and all other patients were grouped into non-ICT treated. PD-L1 protein expression by immunohistochemistry was determined primarily using the 22C3 PD-L1 antibody, and a combined positive score ≥1 was considered positive for survival analysis, as previously described (12). In order to understand whether higher expression of JAK2 and CD274 would have better survival for ICT-treated patients, hazard ratios for survival (and 95% confidence intervals) were computed for each percentile gene expression (where indicated) using the Cox proportional hazards model. Overall survival between groups was compared using the log-rank test.

Figure S1: 9p21.3-related copy-number loss, HPV^{-} HNSC

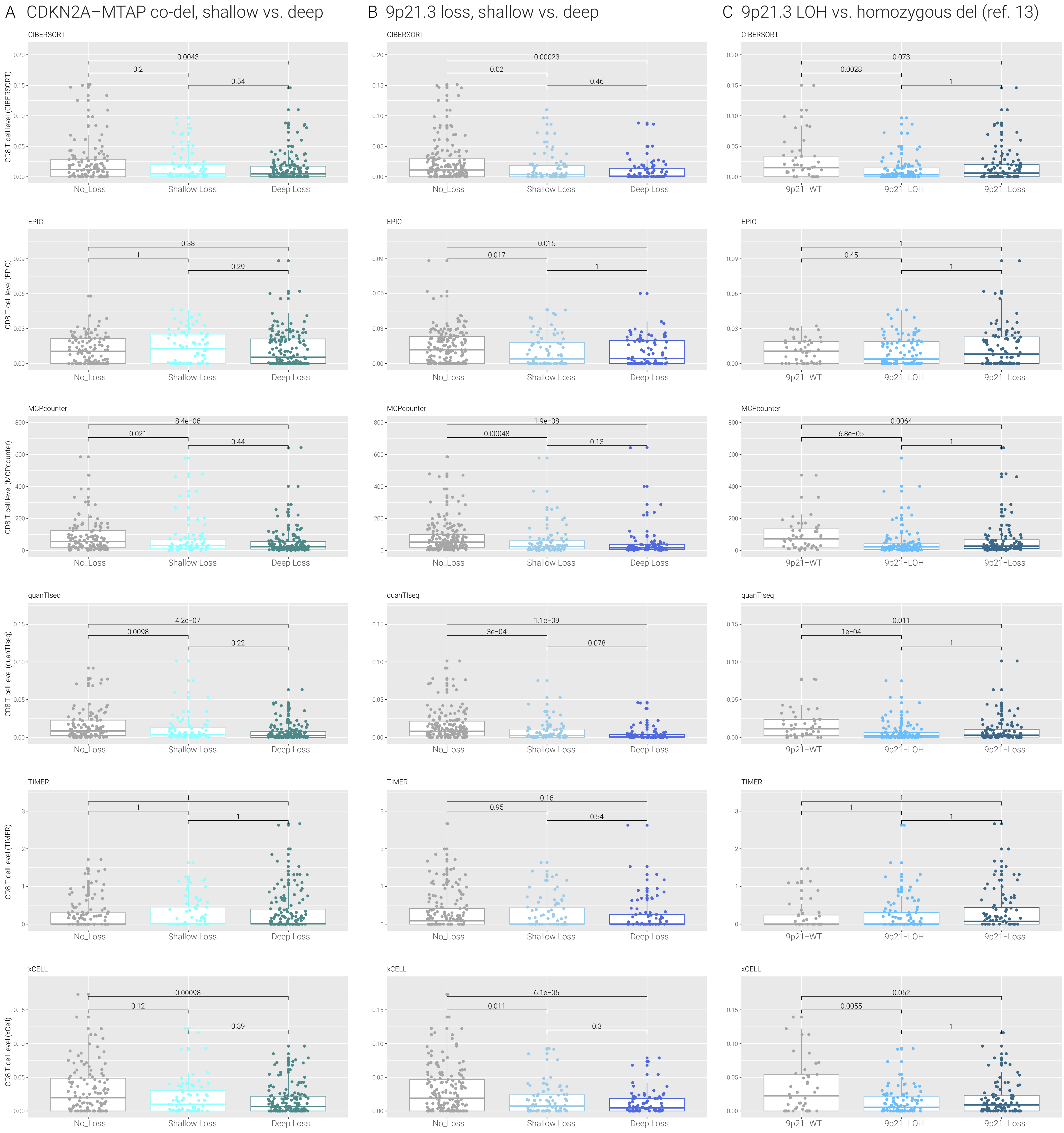

Fig. S1. Difference of CD8 T-cell level between 9p21.3 related SCNA loss in TCGA HPV⁻ HNSC.

A) Box plot represents the CD8 T-cell level among patients showing CDKN2A-MTAP WT, CDKN2A-MTAP shallow loss and CDKN2A-MTAP deep loss by using CIBERSORT, EPIC, MCPcounter, quanTIseq, TIMER and xCell; all p-values are adjusted by false discovery rate.

B) Box plot represents the CD8 T-cell level among patients showing 9p21.3 WT, 9p21.3 shallow loss and 9p21.3 deep loss by using CIBERSORT, EPIC, MCPcounter, quanTIseq, TIMER and xCell; all p-values are adjusted by false discovery rate.

C) Box plot represents the CD8 T-cell level among patients showing 9p21.3 WT, 9p21.3 LOH and 9p21.3 Loss (homozygous loss) by using CIBERSORT, EPIC, MCPcounter, quanTIseq, TIMER and xCell. The category of the patients is determined by (13); all p-values are adjusted by false discovery rate.

Figure S2: 9p and 9p24.1-related shallow vs. deep copy-number loss, HPV⁻ HNSC

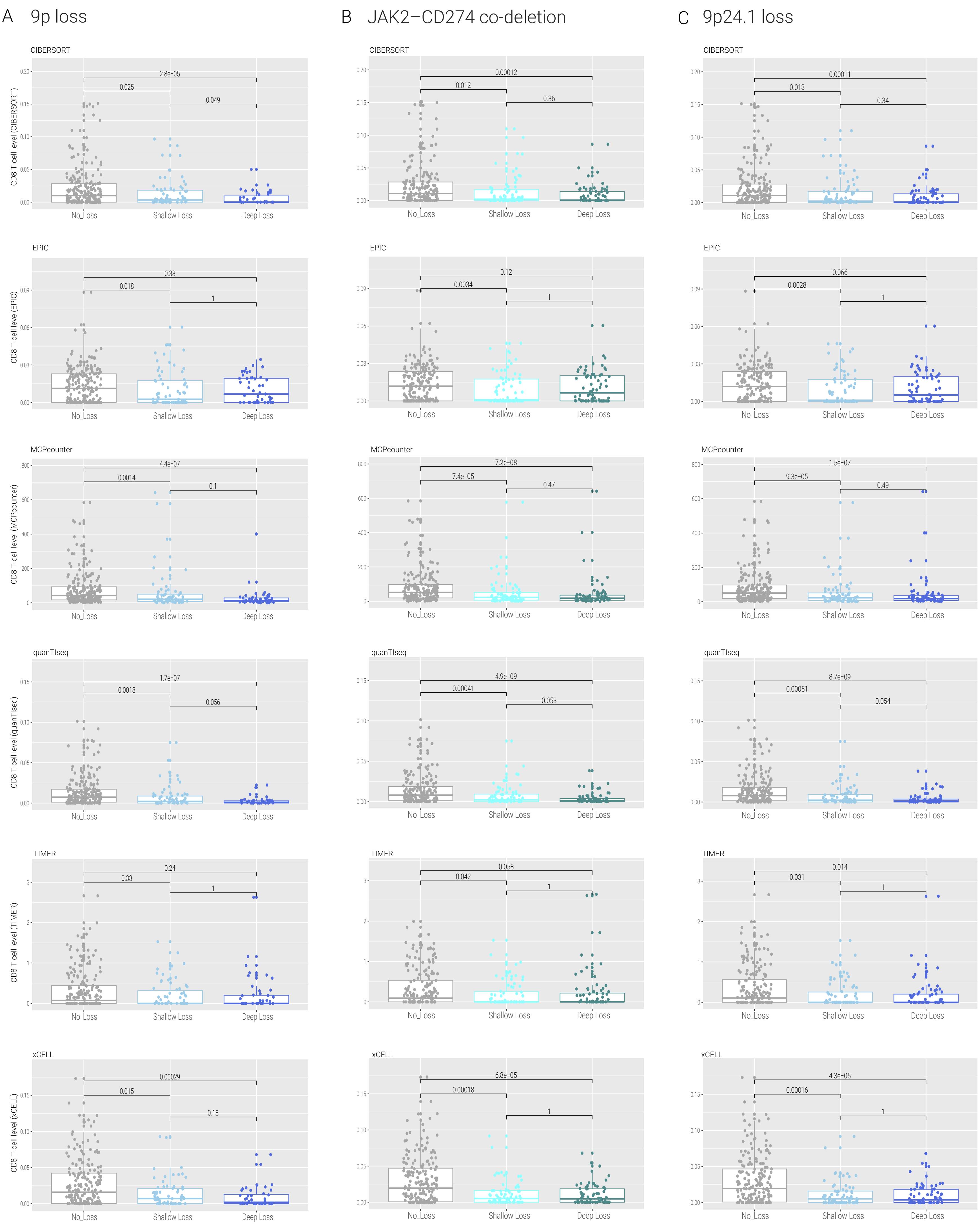

Fig. S2. Difference of CD8 T-cell level between 9p and 9p24.1 related SCNA loss in TCGA HPV⁻ HNSC.

A) Box plot represents the CD8 T-cell level among patients showing 9p WT, 9p shallow loss and 9p deep loss by using CIBERSORT, EPIC, MCPcounter, quanTIseq, TIMER and xCell; all p-values are adjusted by false discovery rate.

B) Box plot represents the CD8 T-cell level among patients showing JAK2-CD274 WT, CDKN2A-MTAP shallow loss and CDKN2A-MTAP deep loss by using CIBERSORT, EPIC, MCPcounter, quanTIseq, TIMER and xCell; all p-values are adjusted by false discovery rate.

C) Box plot represents the CD8 T-cell level among 9p24.1 WT, 9p24.1 shallow loss and 9p24.1 deep loss by using CIBERSORT, EPIC, MCPcounter, quanTIseq, TIMER and xCell; all p-values are adjusted by false discovery rate.

Figure S3: CD8, Immune Score in HPV^{-} HNSC

A Correlation with CD8 T cell (CPTAC)

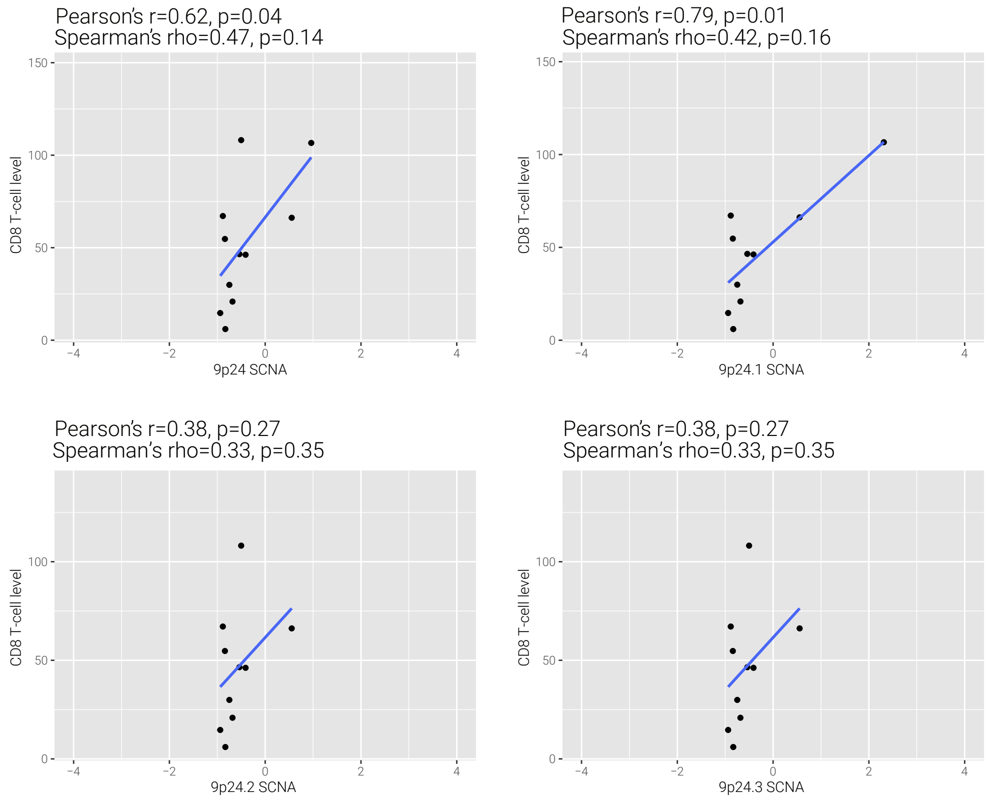

B Correlation with Immune score (CPTAC)

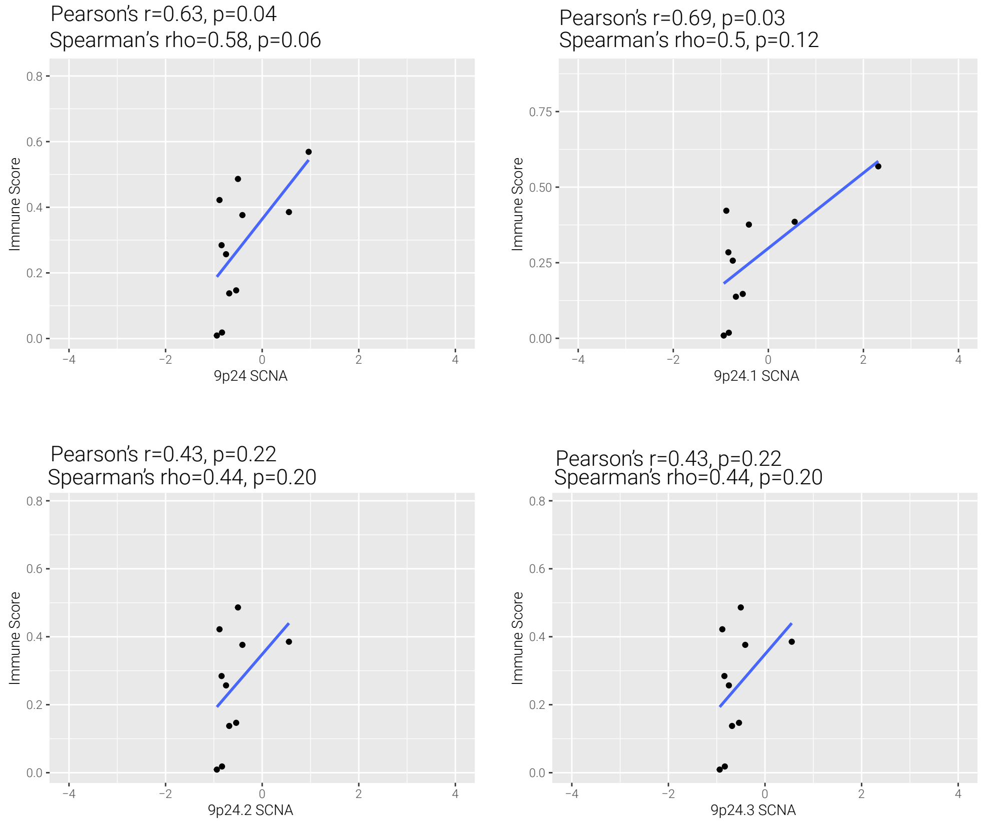

Fig. S3. Correlation between continuous SCNA and immune infiltrates in HPV⁻ HNSC (CPTAC).

A) Dot plots represent correlations between 9p24 focal-level SCNA (3 cytobands 9p24.1, 9p24.2, 9p24.3) and CD8 T-cell level (CPTAC). Both Spearman and Pearson's correlation coefficients and related p-values are indicated in the top of the plot.

B) Dot plots represent correlations between 9p24 focal-level SCNA (3 cytobands 9p24.1, 9p24.2, 9p24.3) and immune score (CPTAC). Both Spearman and Pearson's correlation coefficients and related p-values are indicated in the top of the plot.

Figure S4: 9p24.1 and 9p21.3 SCNA across nine tumor types

A 9p24.1 focal SCNA

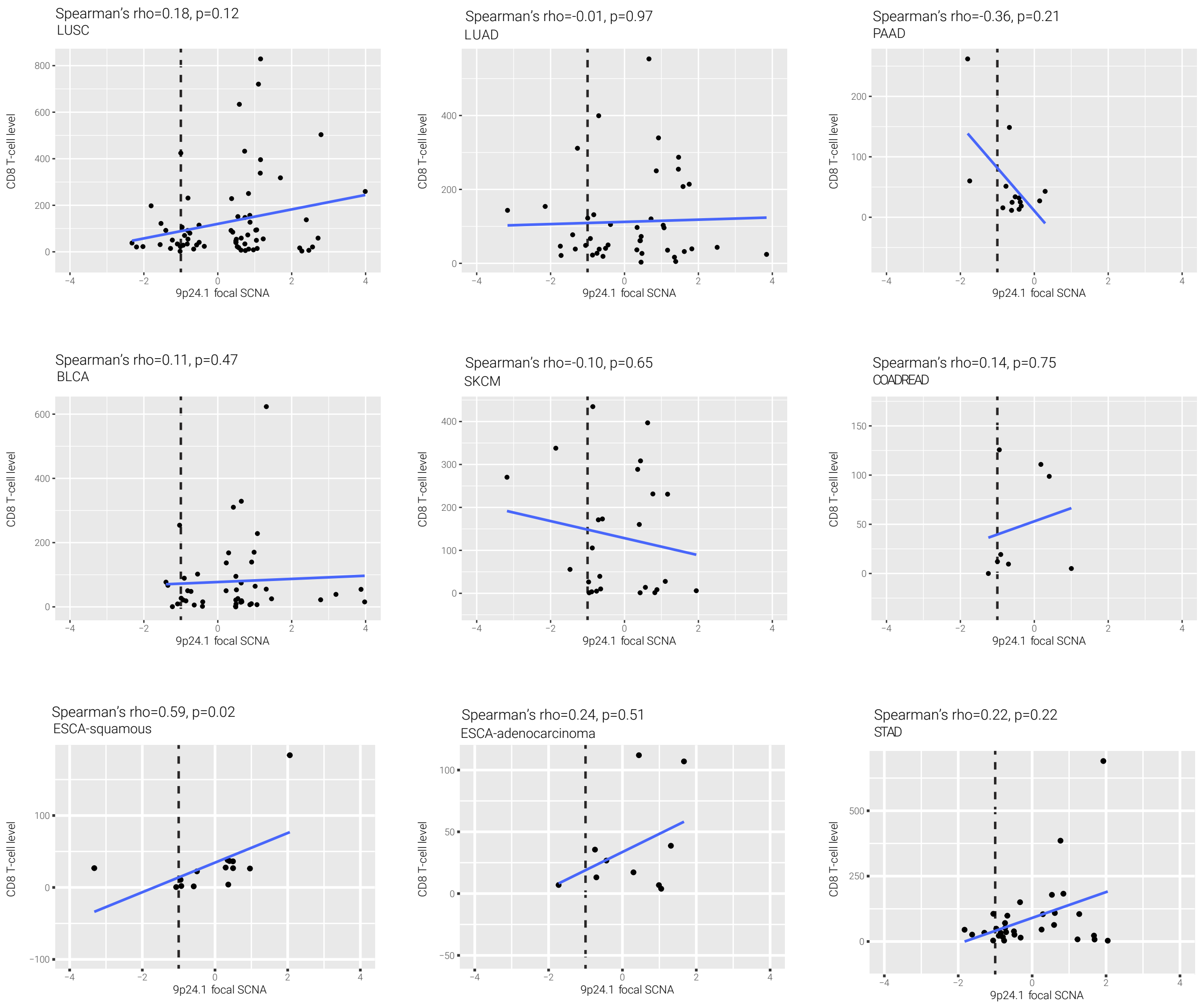

B 9p21.3 focal SCNA

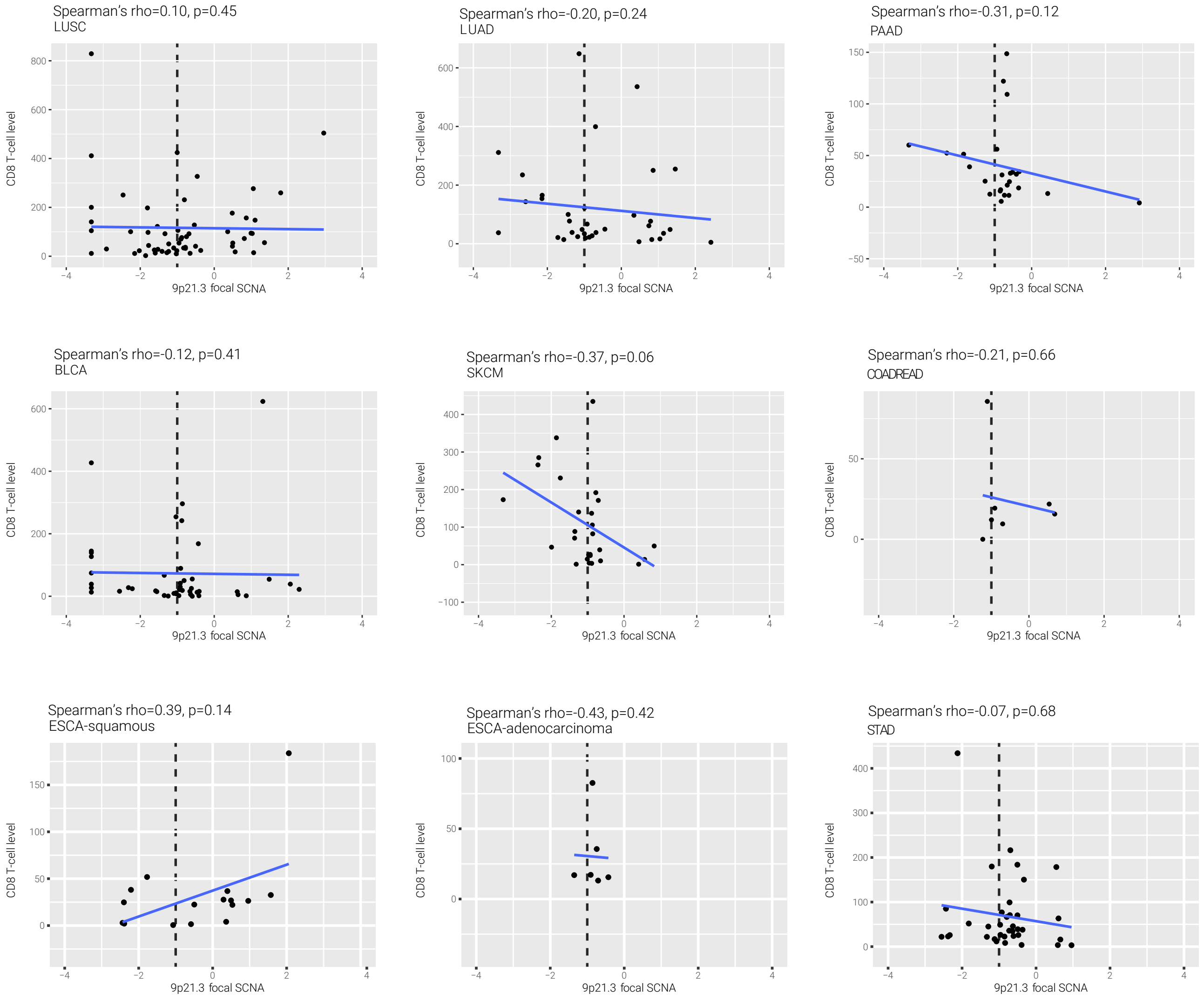

Fig. S4. Correlation between continuous 9p24.1 SCNA or 9p21.3 SCNA and CD8 T-cell level in nine TCGA cancer types.

A) Dot plots represent correlations between 9p24.1 focal-level SCNA and CD8 T-cell in nine different TCGA cancer types, black dot line represents the cutoff of 50% copy number loss (for example, 1 copy loss for diploid genome, 1.5 copy losses for triploid genome and 2 copy losses for tetraploid genome). Spearman correlation coefficients and related p-values are indicated in the top of the plot.

B) Dot plots represent correlations between 9p21.3 focal-level SCNA and CD8 T-cell in nine different TCGA cancer types, black dot line represents the cutoff of 50% copy number loss (for example 1 copy loss for diploid genome, 1.5 copy losses for triploid genome and 2 copy losses for tetraploid genome). Spearman correlation coefficients and related p-values are indicated in the top of the plot.

Figure S5: 9p24.1 gain, Immune Score (pan-TCGA)

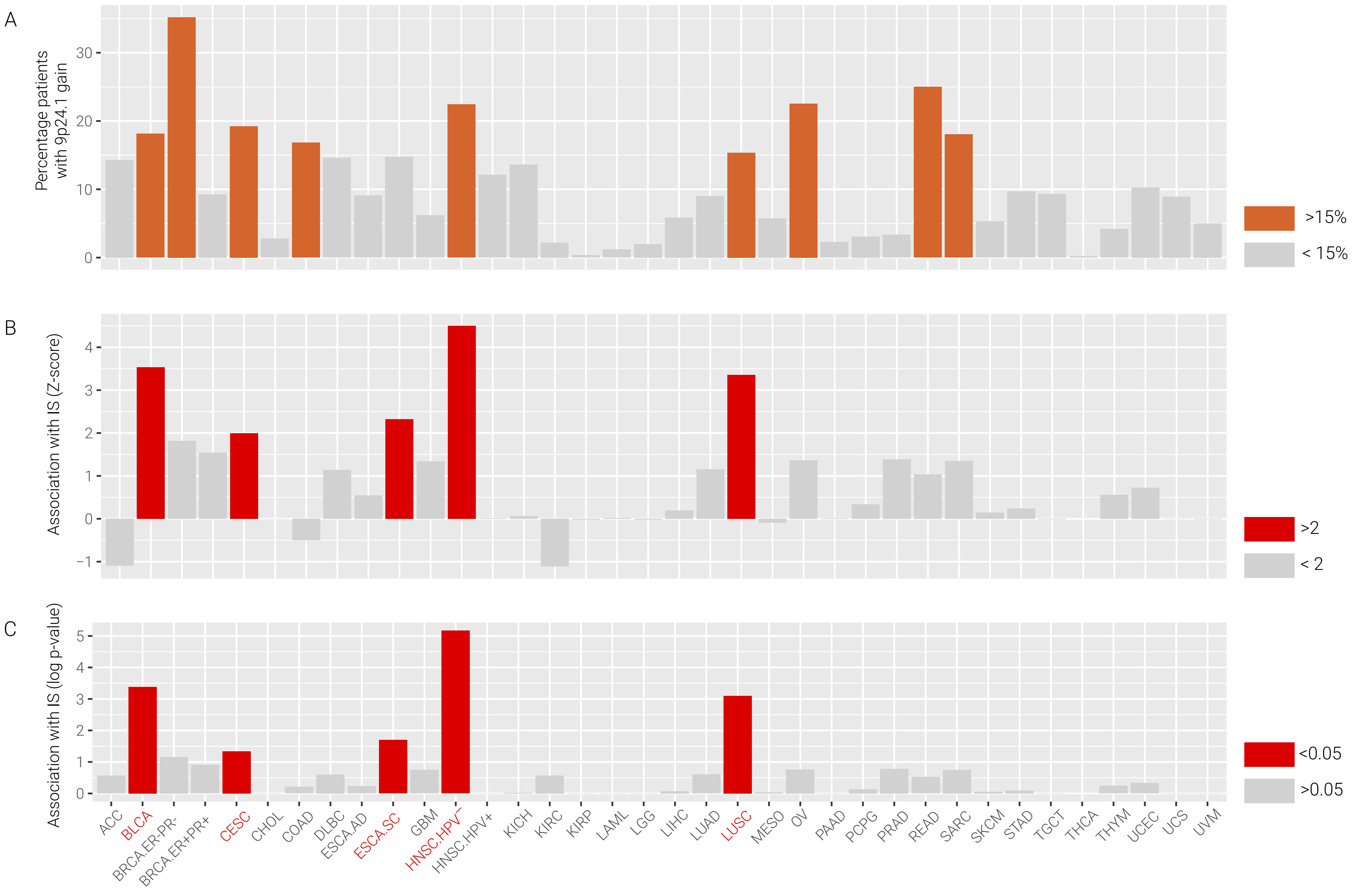

Fig. S5. Association of 9p24.1 gain and immune score across different TCGA cancer types.

A) Bar plot represents the percentage of patients across different TCGA cancer types, patients with percentage higher than 15% are highlighted in orange.

B) Association between 9p24.1 gain and immune score (Z-score) in multivariable logistic regression model (for example, Immune score ~ 9p24.1 gain + SCNA level). Z-scores larger than 2 are highlighted in red.

C) Association between 9p24.1 gain and immune score (log 10 p-value) in multivariable logistic regression model (for example, Immune score ~ 9p24.1 gain + SCNA level). p-value less than 0.05 are highlighted in red.

Figure S6: DNA-RNA Correlations in HPV‾ HNSC

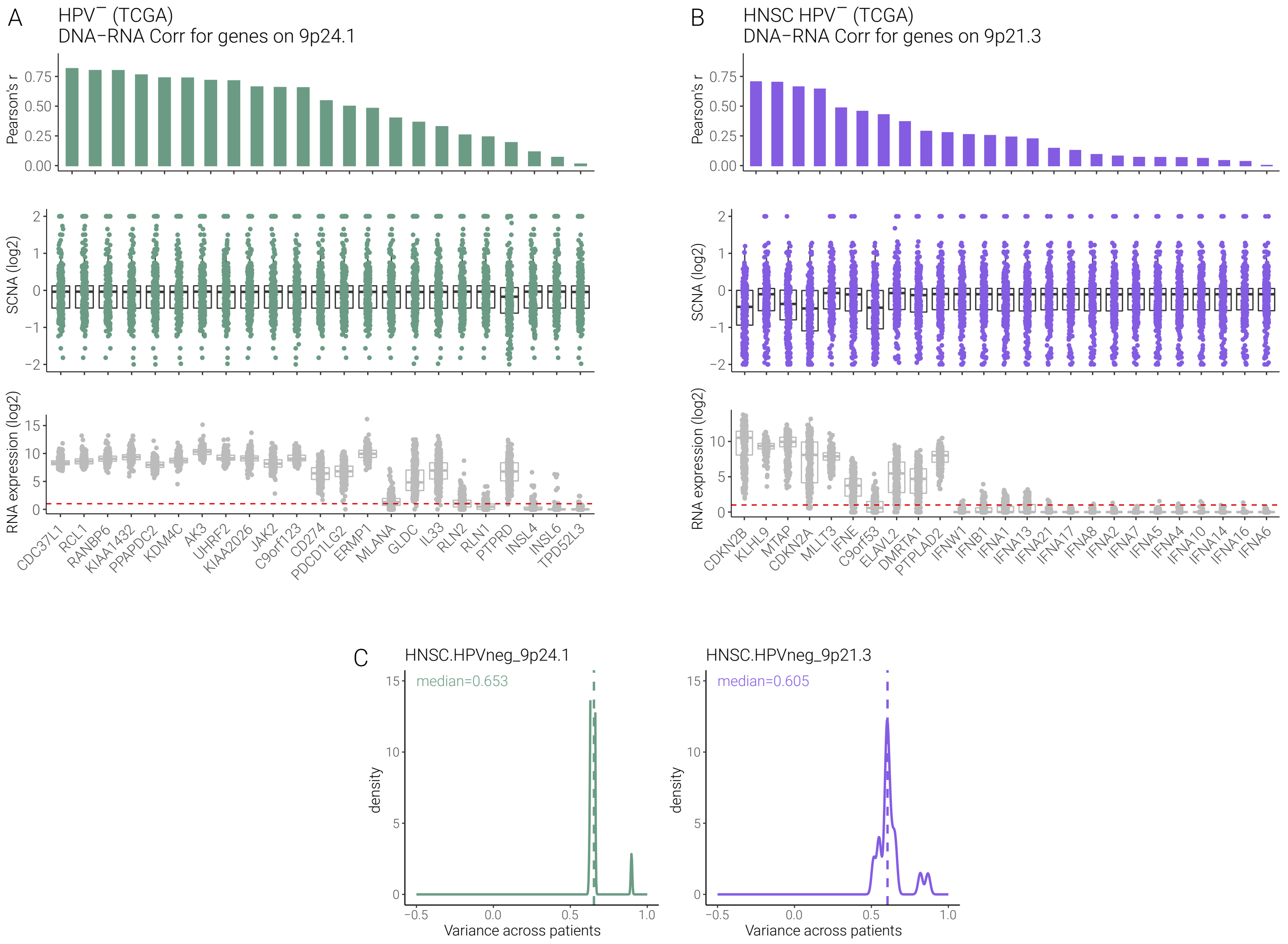

Fig. S6. SCNA and expression for the genes on 9p21.3 and 9p24.1 (TCGA HPV^{-} HNSC).

A) Bar plot represents DNA-RNA correlation (Pearson’s correlation), SCNA level (log2 transformed) and gene expression (log2 transformed) for genes on 9p24.1 (TCGA HPV^{-} HNSC).

B) Bar plot represents DNA-RNA correlation (Pearson’s correlation), SCNA level (log2 transformed) and gene expression (log2 transformed) for genes on 9p21.3 (TCGA HPV^{-} HNSC).

C) Density plot represents the SCNA variance of genes on 9p24.1 region or 9p21.3 region (TCGA HPV^{-} HNSC).

Figure S7: CD274, JAK2, CD274+JAK2 Survival Analyses

A

B

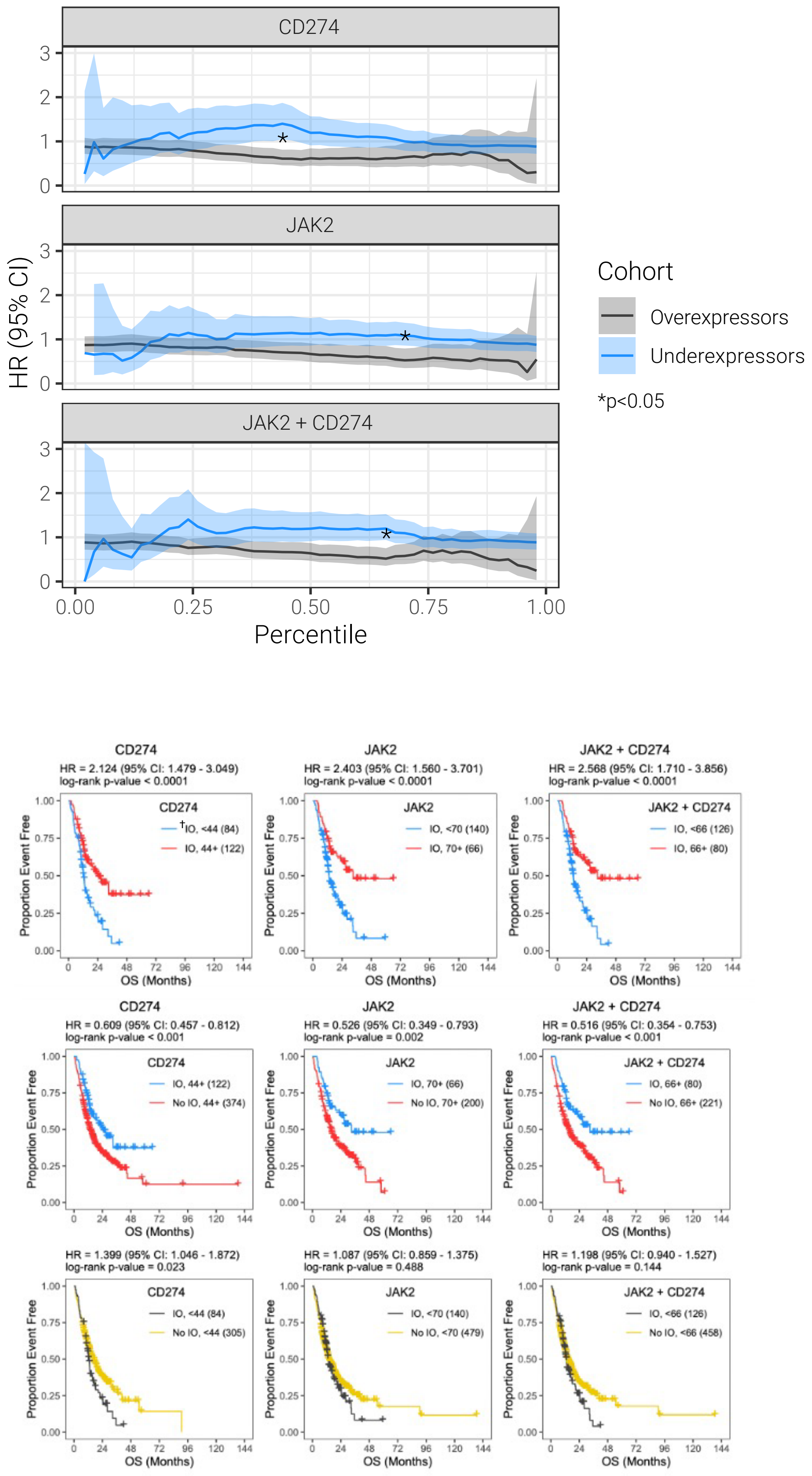

† IO=Immune Checkpoint Therapy (ICT)

Fig. S7. Hazard ratio (HR) for CD274, JAK2 and CD274+JAK2 in HPV⁻ HNSC RWC cohort.

A) Hazard ratio (HR) between over-expressors and under-expressors for gene CD274, JAK2 and JAK2+CD274 at each of the percentile. The optimal percentiles for CD274, JAK2 and JAK2+CD274 is 44th, 70th and 66th, respectively. The asterisk represents the percentile with the largest observed hazard ratio difference between the overexpressor and underexpessor curves.

B) Top row: Kaplan-Meier plots for ICI-treated patients split by observed expression above or below the optimal threshold observed in (A). Middle row: Kaplan-Meier plots for ICT-treated vs non-ICT-treated patients when expression exceeds the optimal threshold. Bottom row: Kaplan-Meier plots for ICT-treated vs non-ICT-treated patients when expression is below the optimal threshold.

Figure S8: 9p24.1 and 9p21.3 Survival Analyses

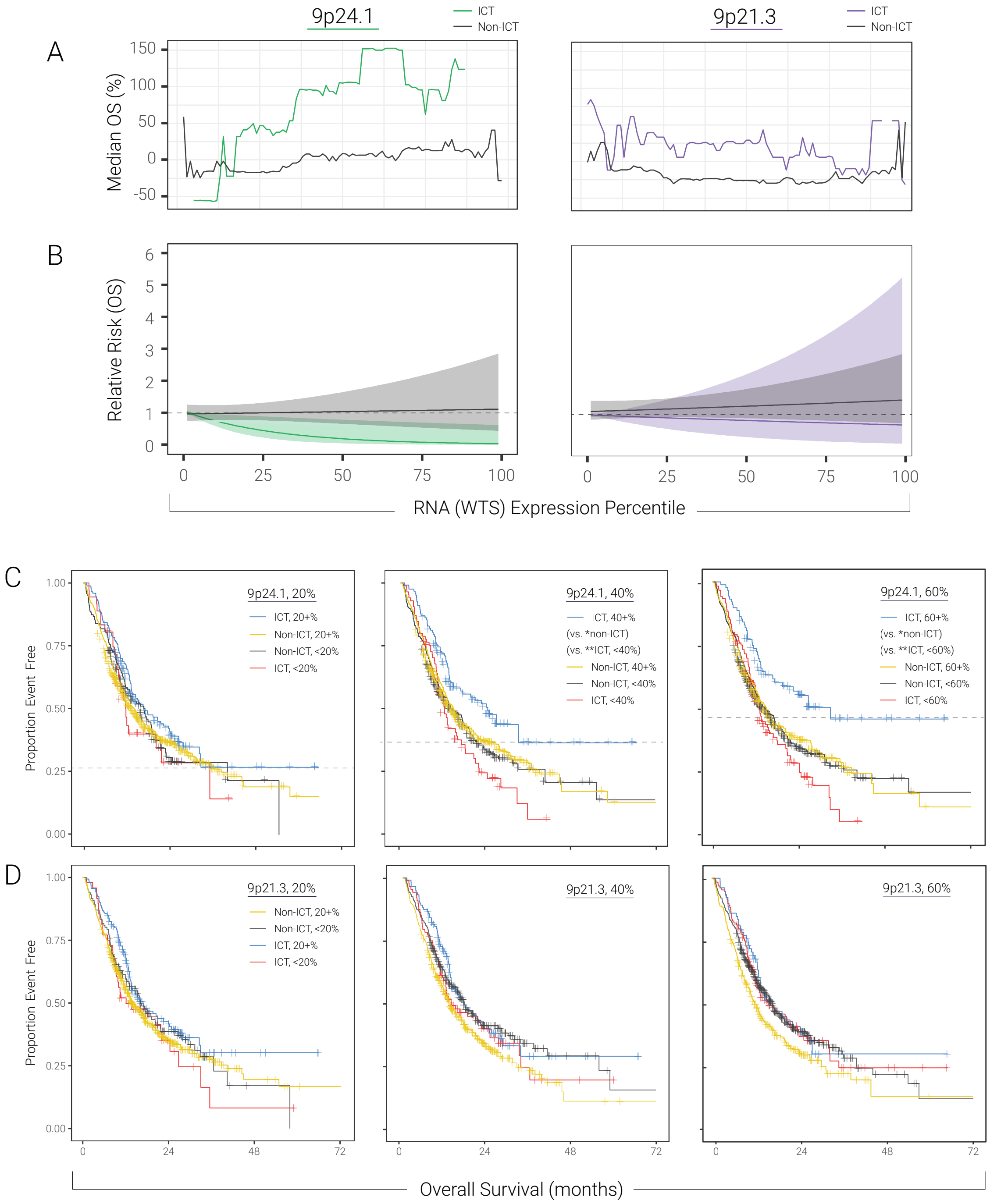

Fig. S8. 9p24.1 and 9p21.3 survival analyses.

A) Median overall survival increase is plotted as a function of expression (moving average of 2% bin size) of all patients independent of treatment or 9p-band status. Survival increase for patient cohorts defined by 9p24.1 expression percentile for patients treated with ICT (green line) or patients treated with any except ICT (black line). Note the peak overall survival at 60th percentile, which could represent small patient numbers in the highest percentiles or a peak biologic effect at that threshold. Survival increase for patient cohorts defined by 9p21.3 expression percentiles for patients treated with ICT (purple line) or patients treated with any except ICT (black line).

B) Cox proportional hazard showing the relative risk at each expression percentile bin. There is a statistical significance to increasing expression of 9p21.3 on survival between the cohort treated with ICT and those not treated with ICT (p-value for expression and interaction < .03). There is no statistical significance to increased or decreased expression of 9p21.3 on survival between the cohort treated with ICT and those not treated with ICT (p-values greater than 0.4). The greater the expression the better the survival for those treated with IO and at low expression, not being treated with ICT confers a survival advantage.

C) Kaplan Meier curves for overall survival, measured from specimen collection date through last as a function of cumulative RNA expression based upon HPV^{-} HNSC, PD-L1+ patients above vs below the 20th, 40th, and 60th percentiles for 9p24.1 treated with and without ICT. *, $p<0.05$ and **, $p<0.01$.

D) Kaplan Meier curves for overall survival, measured from specimen collection date through last as a function of cumulative RNA expression based upon HPV^{-} HNSC, PD-L1+ patients above vs below the 20th, 40th, and 60th percentiles for 9p21.3 treated with and without ICT.

Figure S9: Gene level COX regression on 9p21.3 and 9p24.1 (TCGA HPV⁻ HNSC)

A

Numbers of patients across different stages

Stage I	Stage II	Stage III	Stage IV	Unknown
20	56	65	170	32

B

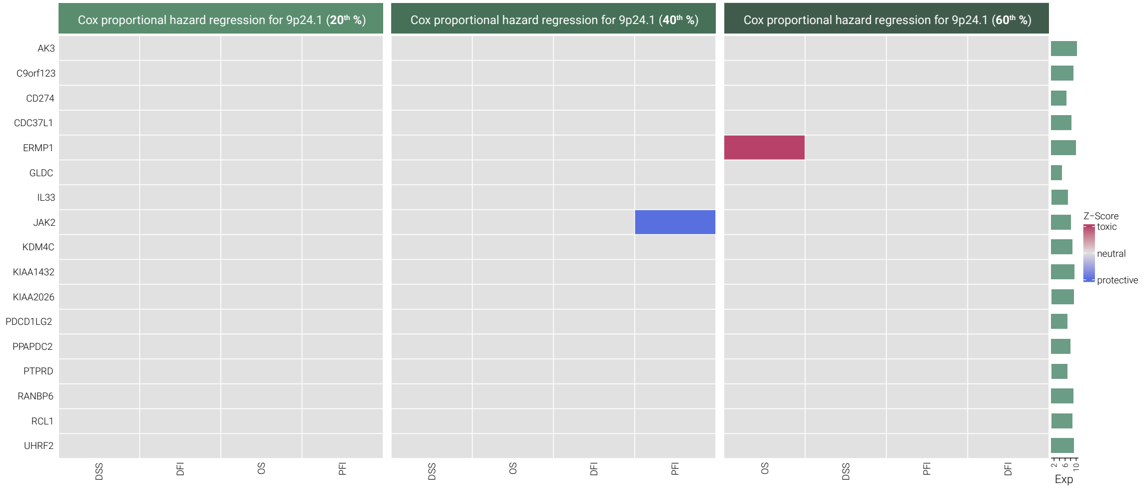

C

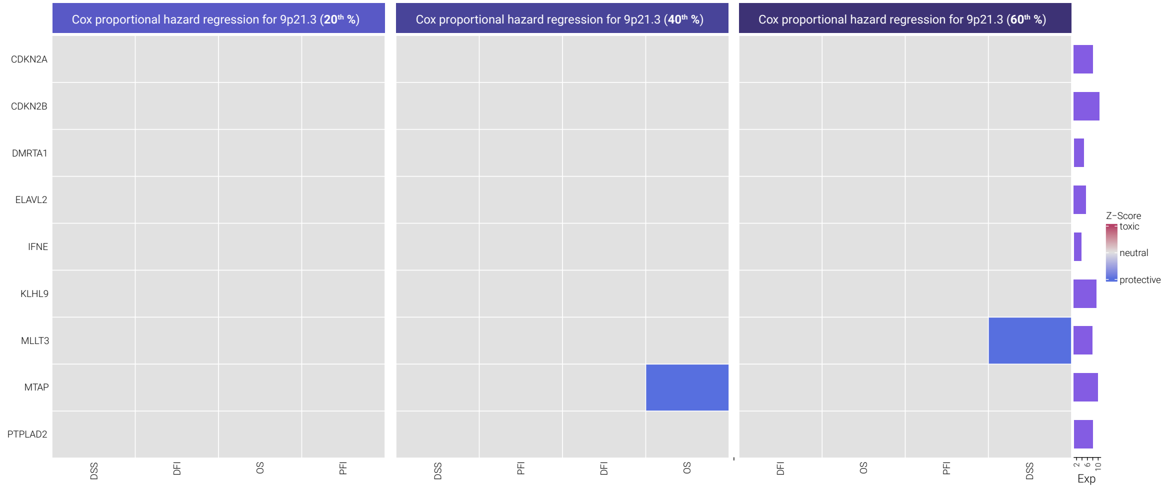

DSS, Disease Specific Survival
OS, Overall Survival

PFI, Progression-Free Interval
DFI, Disease-Free Interval

Fig. S9. Association of 9p24.1 gain and survival in TCGA HPV⁻ HNSC.

A) Number of patients across different stages.

B) Heatmap represents Z-score (Cox Proportional Hazards Regression) after excluding low expression genes on 9p24.1 (purple, from left to right expression at 20th percentile, 40th percentile and 60th percentile) across different survival dataset. Red color represents gene with toxic roles (HR>1) and blue color represents gene with protective roles (HR<1). The bar plot next to heatmap represents the median expression of each gene in TCGA- HPV⁻ HNSC dataset. DSS, disease specific survival; OS, overall survival; PFI, Progression-free interval; DFI, Disease-free interval.

C) Heatmap represents Z-score (Cox Proportional Hazards Regression) after excluding low expression genes on 9p21.3 (green, from left to right expression at 20th percentile, 40th percentile and 60th percentile) across different survival dataset. Red color represents gene with toxic roles (HR>1) and blue color represents gene with protective roles (HR<1). The bar plot next to heatmap represents the median expression of each gene in TCGA- HPV⁻ HNSC dataset. DSS, disease specific survival; OS, overall survival; PFI, Progression-free interval; DFI, Disease-free interval. These findings are consistent with Fig. 3F in recurrent/metastatic HPV⁻ HNSC, showing no significance difference for non-ICT group at 20, 40 and 60 percentiles for the earlier stage TCGA cohort.

Figure S10: HPV^{+} HNSC, 9p24 and 9p21 CD8A, CD8B, and Immune Score

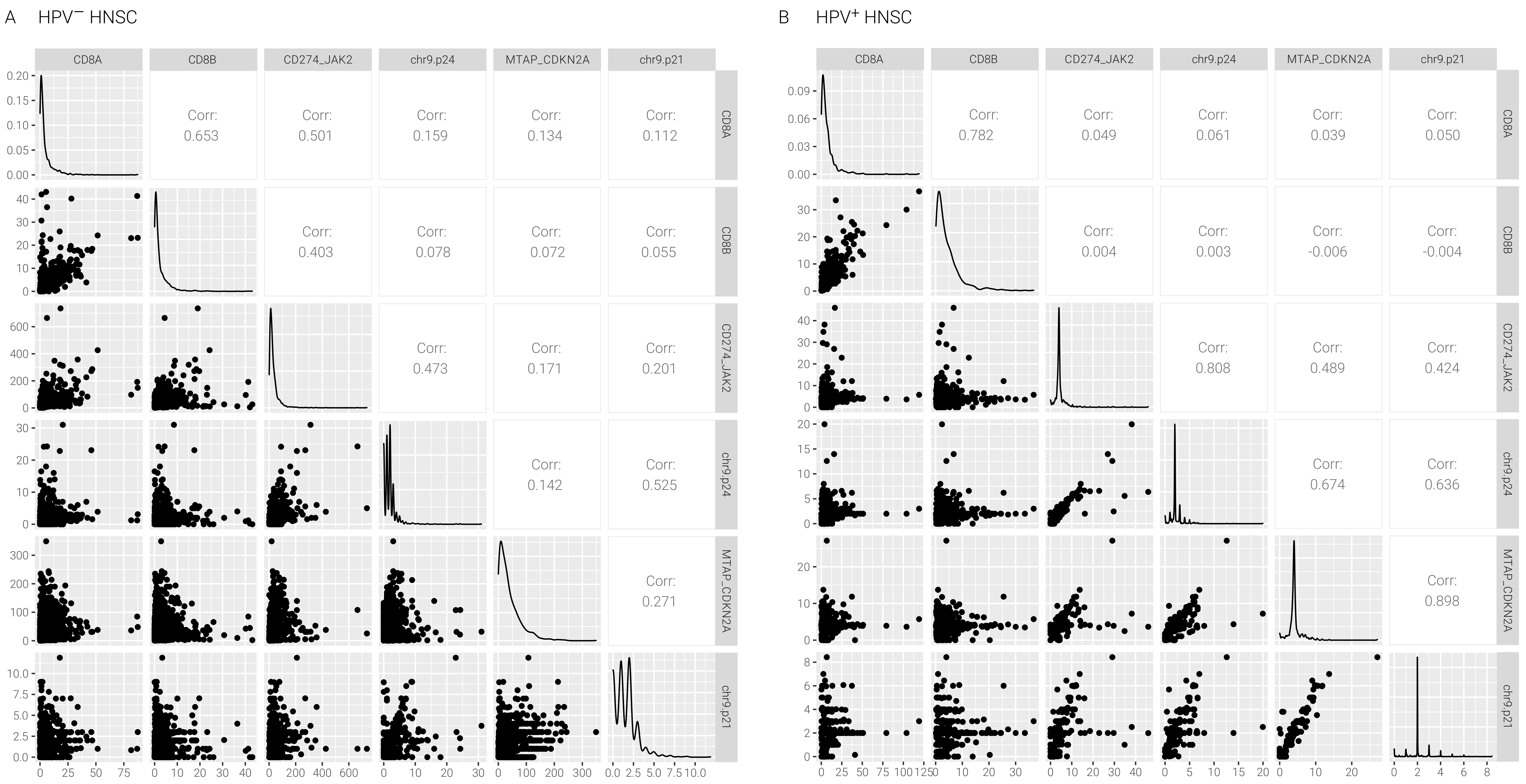

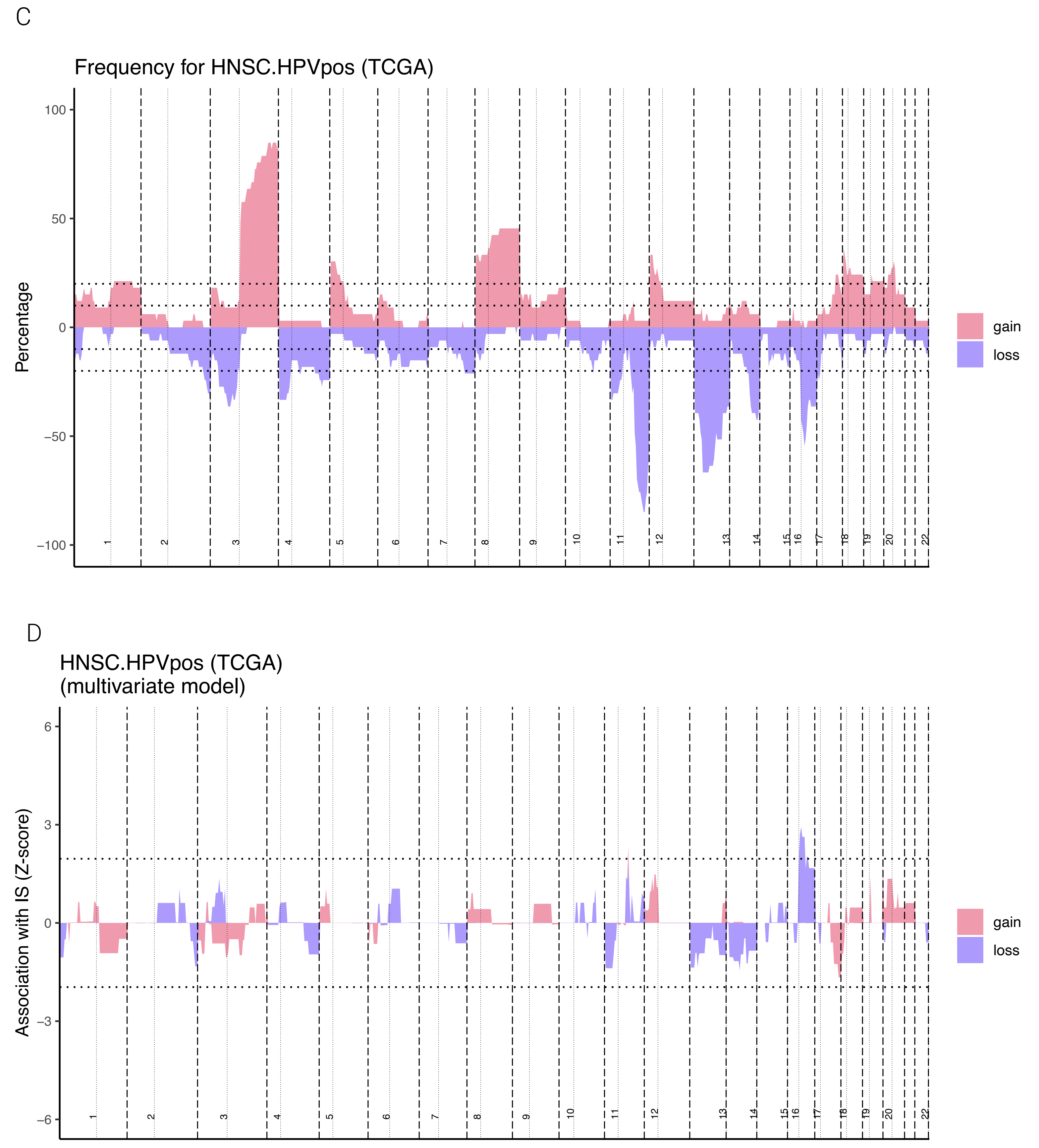

Pearson correlation coefficients:

Biomarker	JAK2 + CD274 HPV^{-}	JAK2 + CD274 HPV^{+}
CD8A	0.501	0.049
CD8B	0.403	0.004

Fig. S10. Association of 9p24.1 gain and CD8 in RWC HPV⁻ and HPV⁺ HNSC.

A) Scatterplot matrix of select RNA expression (CD8A, CD8B, CD274+JAK2, MTAP+CDKN2A) and copy number (chr9p24 and chr9p21) biomarkers among HPV⁻ HNSC cases. Pearson correlation coefficients are annotated in the cells above the diagonal.

B) Scatterplot matrix of select RNA expression (CD8A, CD8B, CD274+JAK2, MTAP+CDKN2A) and copy number (chr9p24 and chr9p21) biomarkers among HPV+ cases. Pearson correlation coefficients are annotated in the cells above the diagonal. We highlight differences in the correlations between CD274+JAK2 and CD8A/B among the HPV+ and HPV⁻ HNSC cohorts in the table below the scatterplot matrices.

C) Area plot represents the percentage of patients with gains (red) or losses (blue) for HPV⁺ HNSC (TCGA) for each chromosomal region. Each chromosome is split by a bold vertical dotted line, the p and q arms are split by the light vertical dotted line. The horizontal dotted line represents 10% and 20% of the patients.

D) Area plot represents the association (Z-score) between gains (red) and losses (blue) and immune score across each chromosome in multivariable logistic regression model (for example, Immune score ~ 9p loss + SCNA level) for HPV⁺ HNSC (TCGA). Each chromosome is split by a bold vertical dotted line, the p and q arms are split by the light vertical dotted line. The horizontal dot line represents p=0.05. Positive Z-score indicates the SCNA is positively associated with the immune score, negative Z-score indicates the SCNA is negatively associated with the immune score.

Datasets S1-S7 (separate files)

Dataset S1. Frequency of loss and association with immune score or CD8 T cells in HPV^{-} HNSC and other TCGA cancers (SCNAs were adjusted by ABSOLUTE)

Dataset S2. Association between CD8 T-cells and SCNAs in a multivariable logistic regression (for example, CD8 T cells ~ 9p arm loss + SCNA level).

Dataset S3. Association between immune parameters and copy-number alterations in different cancer types by a multivariable logistic regression.

Dataset S4. Spearman (Pearson) correlation analysis between CD8 T-cell level and SCNAs on different cytobands.

Dataset S5. Association between CD8+ T-cell and copy-number alterations by a multivariable logistic model (after considering deep loss and shallow loss).

Dataset S6. Spearman correlations between CD8 T-cell expression and expression of genes on 9p24.1 (and 9p21.3) in TCGA HPV^{-} HNSC.

Dataset S7. Additional analyses for survival-related analyses in Figure 3 and Figure S8.

SI References

1. S. L. Carter *et al.*, Absolute quantification of somatic DNA alterations in human cancer. *Nat Biotechnol* **30**, 413-421 (2012).
2. T. Davoli, H. Uno, E. C. Wooten, S. J. Elledge, Tumor aneuploidy correlates with markers of immune evasion and with reduced response to immunotherapy. *Science* **355** (2017).
3. Y. Benjamini, Y. Hochberg, Controlling the False Discovery Rate: A Practical and Powerful Approach to Multiple Testing. *Journal of the Royal Statistical Society: Series B (Methodological)* **57**, 289-300 (1995).
4. E. Becht *et al.*, Estimating the population abundance of tissue-infiltrating immune and stromal cell populations using gene expression. *Genome Biol* **17**, 218 (2016).
5. F. Finotello *et al.*, Correction to: Molecular and pharmacological modulators of the tumor immune contexture revealed by deconvolution of RNA-seq data. *Genome Med* **11**, 50 (2019).
6. A. M. Newman *et al.*, Determining cell type abundance and expression from bulk tissues with digital cytometry. *Nat Biotechnol* **37**, 773-782 (2019).
7. D. Aran, Z. Hu, A. J. Butte, xCell: digitally portraying the tissue cellular heterogeneity landscape. *Genome Biol* **18**, 220 (2017).
8. J. E. Beane *et al.*, Molecular subtyping reveals immune alterations associated with progression of bronchial premalignant lesions. *Nat Commun* **10**, 1856 (2019).
9. E. Talevich, A. H. Shain, T. Botton, B. C. Bastian, CNVkit: Genome-Wide Copy Number Detection and Visualization from Targeted DNA Sequencing. *PLoS Comput Biol* **12**, e1004873 (2016).
10. R. Patro, G. Duggal, M. I. Love, R. A. Irizarry, C. Kingsford, Salmon provides fast and bias-aware quantification of transcript expression. *Nat Methods* **14**, 417-419 (2017).
11. J. Abraham *et al.*, Machine learning analysis using 77,044 genomic and transcriptomic profiles to accurately predict tumor type. *Translational Oncology* **14**, 101016 (2021).
12. K. Emancipator *et al.*, Comparing programmed death ligand 1 scores for predicting pembrolizumab efficacy in head and neck cancer. *Mod Pathol* **34**, 532-541 (2021).
13. G. Han *et al.*, 9p21 loss confers a cold tumor immune microenvironment and primary resistance to immune checkpoint therapy. *Nat Commun* **12**, 5606 (2021).
